# Stretchable and self-healable hydrogel artificial skin

**DOI:** 10.1093/nsr/nwab147

**Published:** 2021-08-14

**Authors:** Bin Xue, Hui Sheng, Yongqiang Li, Lan Li, Weishuai Di, Zhengyu Xu, Linjie Ma, Xin Wang, Haoting Jiang, Meng Qin, Zhibo Yan, Qing Jiang, Jun-Ming Liu, Wei Wang, Yi Cao

**Affiliations:** National Laboratory of Solid State Microstructures, Department of Physics, Nanjing University, Nanjing 210093, China; National Laboratory of Solid State Microstructures, Department of Physics, Nanjing University, Nanjing 210093, China; National Laboratory of Solid State Microstructures, Department of Physics, Nanjing University, Nanjing 210093, China; State Key Laboratory of Pharmaceutical Biotechnology, Department of Sports Medicine and Adult Reconstructive Surgery, Drum Tower Hospital Affiliated to Medical School of Nanjing University, Nanjing 210008, China; National Laboratory of Solid State Microstructures, Department of Physics, Nanjing University, Nanjing 210093, China; National Laboratory of Solid State Microstructures, Department of Physics, Nanjing University, Nanjing 210093, China; National Laboratory of Solid State Microstructures, Department of Physics, Nanjing University, Nanjing 210093, China; National Laboratory of Solid State Microstructures, Department of Physics, Nanjing University, Nanjing 210093, China; National Laboratory of Solid State Microstructures, Department of Physics, Nanjing University, Nanjing 210093, China; National Laboratory of Solid State Microstructures, Department of Physics, Nanjing University, Nanjing 210093, China; National Laboratory of Solid State Microstructures, Department of Physics, Nanjing University, Nanjing 210093, China; State Key Laboratory of Pharmaceutical Biotechnology, Department of Sports Medicine and Adult Reconstructive Surgery, Drum Tower Hospital Affiliated to Medical School of Nanjing University, Nanjing 210008, China; National Laboratory of Solid State Microstructures, Department of Physics, Nanjing University, Nanjing 210093, China; National Laboratory of Solid State Microstructures, Department of Physics, Nanjing University, Nanjing 210093, China; Institute for Brain Sciences, Nanjing University, Nanjing 210093, China; National Laboratory of Solid State Microstructures, Department of Physics, Nanjing University, Nanjing 210093, China; Institute for Brain Sciences, Nanjing University, Nanjing 210093, China; Chemistry and Biomedicine Innovation Center, Nanjing University, Nanjing 210093, China

**Keywords:** hydrogel, capacitive sensor, stretchability, self-healing, self-assembly

## Abstract

Hydrogels have emerged as promising materials for the construction of skin-like mechanical sensors. The common design of hydrogel-based artificial skin requires a dielectric sandwiched between two hydrogel layers for capacitive sensing. However, such a planar configuration limits the sensitivity, stretchability and self-healing properties. Here, we report the design of single-layer composite hydrogels with bulk capacitive junctions as mechanical sensors. We engineer dielectric peptide-coated graphene (PCG) to serve as homogenously dispersed electric double layers in hydrogels. Any mechanical motions that alter the microscopic distributions of PCG in the hydrogels can significantly change the overall capacitance. We use peptide self-assembly to render strong yet dynamic interfacial interactions between the hydrogel network and graphene. The resulting hydrogels can be stretched up to 77 times their original length and self-heal in a few minutes. The devices can effectively sense strain and pressure in both air and aqueous environments, providing tremendous opportunities for next-generation iontronics.

## Teaser

Single-layer composite hydrogels with bulk capacitive junctions were engineered as highly stretchable, self-healing, and printable artificial skins for multi-modal mechanical sensing by dispersing dielectric peptide-coated graphene in the gel matrix.

## INTRODUCTION

Human skin combines outstanding mechanical properties with multifunctional sensing abilities. These characteristics have inspired the development of artificial skin-like materials for flexible electronics with various advanced applications in soft robotics [1,2], human-machine interfaces [3,4] and wearable electronic devices [5,6]. Initially, artificial skin was made of elastomers embedded with stretchable electronic conductors, such as carbon black [7], metals [8,9], conductive polymers [10,11], carbon nanotubes [12,13], graphene [14,15] and Mxenes [16,17]. The mechanical sensing is based on the change of conductivity. Recently, ionic conductors, such as hydrogels, have attracted considerable research interest because of their high biocompatibility, similar softness to human tissues, great stretchability and water tolerance [18,19]. Some hydrogel-based artificial skins still rely on change of electrical conductivity for mechanical sensing [20,21]. However, the electrical properties of the conductive composites strongly depend on the density of the conductive networks. Increasing the conductivity by increasing the concentration of conductive fillers inevitably compromises the stretchability. Alternatively, hydrogel-based artificial skins can be engineered based on a capacitive sensing mechanism. They comprise a dielectric elastomer sandwiched between two conductive hydrogel layers, and pressure and strain can be detected by a change in capacitance [22,23].

Despite the tremendous success of such designs, creating hydrogel artificial skin that is stretchable and self-healable so that it can repair unexpected internal or external damage and recover critical functions similar to human skin remains challenging [24]. First, high stretchability and fast self-healing are often conflicting requirements [25,26]. Hydrogels made of chemically cross-linked networks are stable but lack a mechanism to self-heal. Although hydrogels cross-linked by reversible physical interactions can self-heal, they are mechanically compliant and cannot tolerate large strain. Moreover, the planar configuration of hydrogel artificial skin has certain limitations. Without strong interfacial bonding, the hydrogel and the elastomer layers are susceptible to delamination under multiple strain cycles because of their different chemical and mechanical properties [2]. Achieving simultaneous self-healing of the hydrogel and the elastomer layers to fully recover the functionalities of hydrogel artificial skin is almost impossible [27]. The relatively small electric double layer area also limits the sensitivity [28].

Here, we propose a single-layer hydrogel artificial skin, termed ‘SHARK’, which combines high stretchability, self-healing properties and ultrasensitive mechanical sensing. In sharp contrast to traditional artificial skin of sandwich structures and integrated mechanical sensing, SHARK can be considered a bulk junction of hydrogel capacitors, analogous to the structure of bulk polymeric solar cells [29]. The electronic conducting layers are dispersed in the gel matrix of SHARK, forming distributed yet interconnected mechano-sensors akin to human skin. We develop an interfacial self-assembly technology to render strong yet dynamic bonding between conducting layers and the hydrogel matrix. Therefore, SHARK can be stretched up to a 7700% strain and maintain linear sensing up to 2600%. Such design also allows simultaneous self-healing of mechanical and electrical properties after damage. Moreover, the large electric double layer area enables ultrasensitive sensing of pressure and strain using SHARK. We expect that such a novel design can greatly improve the performance of hydrogel-based artificial skin and enable the construction of complicated hydrogel-based wearable devices.

## RESULTS AND DISCUSSION

### Design and molecular engineering of SHARK

**Figure 1.**
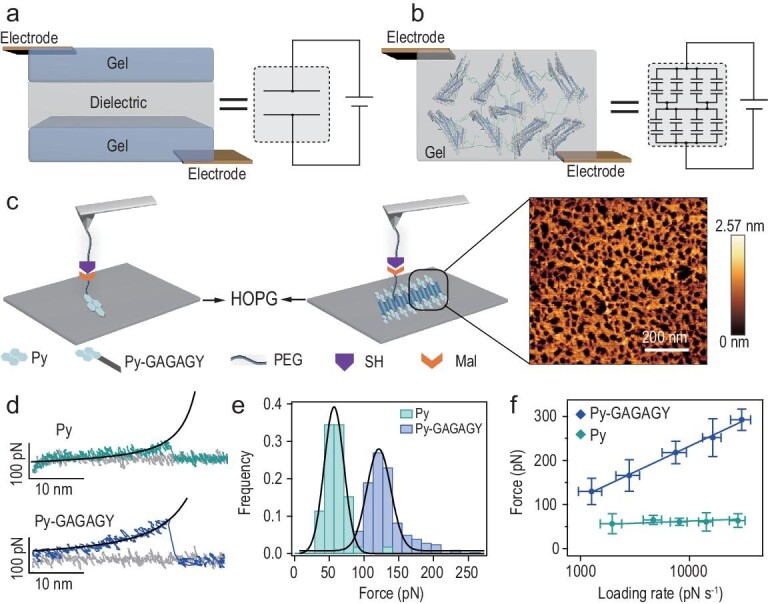
Design and molecular engineering of SHARK. (a and b) Comparison of the structures and equivalent electric circuits of typical sandwich-shaped hydrogel-based artificial skin (a) and SHARK (b). SHARK comprises a bulk junction of hydrogel capacitors made of dielectric peptide-coated conductive graphene layers dispersed in the hydrogel matrix. The strong yet dynamic interactions between the peptide and the graphene layers endow SHARK with high mechanical stability and great self-healing properties. (c) AFM-based single-molecule force spectroscopy experiments are used to characterize the interfacial bonding between graphene and the polymeric hydrogel network through self-assembled peptide layers. The experimental schemes for measuring the adsorption forces between Py or Py-GAGAGY and the graphite surface are shown in the left and middle panels. Py or Py-GAGAGY is allowed to adsorb on HOPG and is then picked up by a PEG-functionalized cantilever tip through maleimide-thiol chemistry. Prior to the single-molecule experiment, Py-GAGAGY self-assembled into a fibrous network on the HOPG surface, as confirmed by AFM imaging (right). (d) Typical force-extension curves at a pulling speed of 400 nm s^−1^. Black lines correspond to worm-like chain fitting to the retraction traces. The heights of the peak correspond to the detachment forces. (e) Rupture force histograms for Py and Py-GAGAGY from graphite surfaces at a pulling speed of 400 nm s^−1^. The Gaussian fittings show average dissociation forces of 56.5 ± 22.5 pN (n = 118) and 129.8 ± 29.7 pN (n = 207). (f) Dynamic force spectroscopy experiments for the dissociation of adsorbed Py and Py-GAGAGY from graphite at various force loading rates. The dissociation of Py from graphite is almost loading rate independent, indicating that the binding and unbinding are in fast equilibrium. However, the dissociation of Py-GAGAGY is a nonequilibrium process and is strongly dependent on the loading rate. Fitting the experimental results to the Bell-Evans model (solid lines) yields a spontaneous dissociation rate of 2.0 s^−1^ and a potential width of 0.08 nm. Self-assembly of Py-GAGAGY greatly enhances the binding strength of Py with graphite and shifts the binding/unbinding equilibrium to a time scale slower than the force loading rates in the experiments.

Unlike the traditional sandwich sensor with conductive layers acting as the electrode slab (Fig. 1a), SHARK is composed of peptide-coated graphene (PCG) sheets dispersed in a polyacrylamide hydrogel network (Fig. 1b, left). The adjacent graphene sheets can act as the conductive layers of a microcapacitor, and the peptide coated on the graphene sheets as well as the polymer between the graphene sheets act as the dielectric of the microcapacitor. The whole system can be considered a bulk capacitor junction formed by series-parallel connection of numerous microcapacitors (Fig. 1b, right). As such, SHARK features larger equivalent electric double layer areas and thus higher sensitivities than planar-shaped hydrogel sensors. Any mechanical motions that affect the microscopic distributions of PCG in the hydrogels can significantly change the overall capacitance. The most important part of the design is the interface between graphene and the hydrogel network. Each side of the graphene is coated by a layer of self-assembled peptides. Because the dielectric constant of the peptide is much lower than that of water [30], this can further increase the capacitive sensing performance. Moreover, self-assembly of the peptides greatly enhances the interfacial bonding strength but does not affect the dynamic and reversible properties. This is essential for achieving combined high stretchability and fast self-healing.

Specifically, the peptide sequence is GAGAGY (G = glycine, A = alanine and Y = tyrosine). GAGAGY is a self-assembling motif derived from *Bombyx mori* silk protein and can typically form β-sheet structures [31]. A pyrene group (Py) was introduced to the N-terminus of the peptide, which can bind graphene through hydrophobic interactions and π–π stacking (Figs S1a and S2) [32]. The peptide is denoted Py-GAGAGY hereafter. Self-assembly of the peptide on highly oriented pyrolytic graphite (HOPG) surfaces was studied by atomic force microscopy (AFM) imaging (Figs 1c right and S3a–c). A fibrous peptide network could be clearly observed. The width of the peptide fiber was ∼1.2 nm (Fig. S3c), well matching the length of the peptide. This suggested that the peptides are arranged as parallel β-sheets perpendicular to the fiber axis. The formation of β-sheet structures was further confirmed by infrared spectroscopy (Fig. S4a) [33]. We next tested whether the formation of a fibrous peptide network could enhance the bonding of the peptide to graphite surfaces so that we could use this strong interaction to mechanically exfoliate high-quality graphene sheets and construct stretchable hydrogels. Our AFM-based single-molecule force spectroscopy experiments indicated that the dissociation forces between the pyrene and graphite surfaces increased from 56.5 ± 22.5 pN for Py to 129.8 ± 29.7 pN for Py-GAGAGY at a pulling speed of 400 nm s^−1^ (Fig. 1c–e). The rupture forces became even higher at higher force-loading rates, providing a unique mechanism to resist mechanical damage at large strain rates. Dynamic force spectroscopy experiments suggested that this was mainly achieved through shortening of the potential width for dissociation, while the dissociation rate remained very fast (2.0 s^−1^, Fig. 1f). A shorter potential width implies more cooperative bonding. We proposed that in self-assembled peptide fibers, interactions between neighboring peptides provide additional stabilization forces. This stabilization effect is more obvious at a timescale faster than the intrinsic dynamics of the peptide-peptide interactions (corresponding to higher strain rates) but vanished at longer timescales (corresponding to lower strain rates). These results confirmed that Py-GAGAGY can efficiently self-assemble on the surface of graphite, leading to strong yet dynamic interfacial bonding.

### Production of PCG via aqueous exfoliation

**Figure 2.**
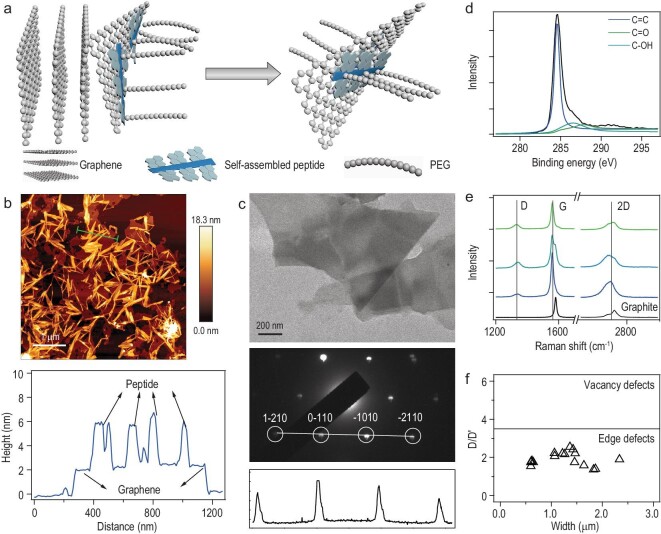
Characterization of PCG prepared via aqueous exfoliation. (a) Scheme of the ultrasound-assisted mechanical exfoliation of graphite in the presence of self-assembling Py-GAGAGY peptides. (b) AFM topographic image (top) and height profile (bottom) of the self-assembled peptide fibers on an exfoliated graphene surface. (c) TEM image (top), electron diffraction pattern (middle) and diffracted intensity (bottom) taken along the 1$\bar{2}$10 to $\bar{2}$210 axis of the graphene after removing the peptide using dialysis. (d) C 1s XPS spectrum of the graphene. The peak areas of C=C, C−OH and C=O are 9436, 918 and 606 a.u., respectively. The low oxygen content indicates the high quality of the produced graphene. (e) Representative Raman spectroscopy of PCG and graphite (excited at 532 nm). (f) Distributions of D/D’ and width of PCG based on Raman spectroscopy. Low D/D’ ratios indicate that the produced graphene only contains a few edge defects and no vacancy or sp^3^ defects.

Next, we prepared PCG by direct aqueous exfoliation of graphite via ultrasonication. Inspired by previous studies using proteins and peptides as dispersants to obtain high-quality graphene [34], we developed a method to produce Py-GAGAGY dispersed graphene by exfoliating graphite in Py-GAGAGY aqueous solutions via ultrasonication (Fig. 2a). Instead of using Py-GAGAGY alone, we mixed it with the peptide of a C-terminal polyethylene glycol (mPEG-NH2, 10 kDa) extension (Py-GAGAGY-mPEG, Fig. S5). The mixed Py-GAGAGY and Py-GAGAGY-mPEG can co-assemble into fibrous peptide networks on the HOPG surfaces (Figs S3d and S4a). In the aqueous exfoliation process, Py-GAGAGY and Py-GAGAGY-mPEG can serve as biosurfactants to stabilize the graphene nanosheets. More importantly, the mPEG attached to the peptide nanofibers can significantly increase the solvodynamic dragging forces and boost the exfoliation efficiency (Fig. 2a). The maximal yield is determined by the interplay of the surface binding forces and the solvodynamic dragging forces. The optimal yield appeared at the Py-GAGAGY: Py-GAGAGY-mPEG ratio of 10 : 1 (Fig. S6). The highest concentration of the produced PCG solutions can reach ∼640 μg mL^−1^ (64% yield) for the initial graphite concentration of 1.0 mg mL^−1^, higher than those produced with the assistance of other biomolecules (Table S1). Another remarkable feature of PCG is its long-term stability, which ensures high mechanical and electrical stability of SHARK. The concentration of the as-prepared PCG solutions showed no obvious decrease in one month (Fig. S7a) and decreased by less than 30% after 90 days at room temperature, as estimated by UV spectroscopy (Fig. S7b and c). Moreover, the PCG solutions can be concentrated to 6 mg mL^−1^ without aggregation. However, as the interfacial bonding between the peptide and graphene is dynamic and reversible, the peptide may occasionally dissociate from the graphene surfaces, leading to slight aggregation of graphene during the long-term storage.

We then estimated the layer number, width and defects of the PCG using AFM, transmission electron microscopy (TEM), X-ray photoelectron spectroscopy (XPS) and Raman spectroscopy (Fig. 2b–e). Our characterizations confirmed the successful production of few-layer graphene sheets with an average of 1.9 ± 0.3 layers (mean ± standard error of the mean) and lateral sizes of 2.0 ± 0.4 μm for the Py-GAGAGY : Py-GAGAGY-mPEG ratio of 10 : 1, at which the highest production yield was achieved (Figs 2b–e and S8). The graphene sheets were also of high quality with only edge defects and almost no vacancy or sp^3^ defects based on Raman spectroscopy (Fig. 2f). The chemical and physical properties of the produced PCG did not greatly vary at different Py-GAGAGY : Py-GAGAGY-mPEG ratios (Fig. S8). To integrate the graphene sheet with the polymer network in the hydrogel preparation step, we also introduced a C=C bond to the peptide by adding a C-terminal acrylate-conjugated lysine residue (named Py-GAGAGYK-ACLT, Fig. S1b and c). Py-GAGAGYK-ACLT showed similar self-assembly properties to Py-GAGAGY (Figs S4b and S9), and this modification did not affect the preparation of PCG sheets.

### Preparation and characterization of SHARK

**Figure 3.**
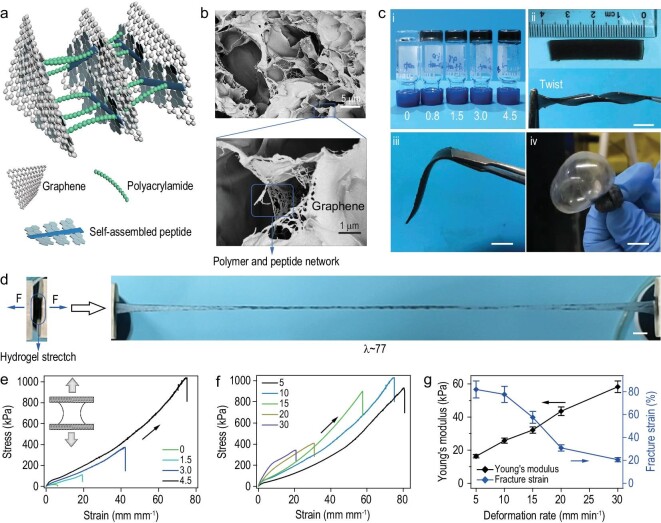
Design, characterization and mechanical properties of SHARK. (a) Schematic of the SHARK hydrogel network. PCG nanosheets are linked to each other via in situ copolymerization of acrylamide and acrylate-modified Py-GAGAGY peptide. (b) SEM images of the porous structures of the hydrogel. The self-assembled peptide networks can be clearly observed on the graphene sheets in the enlarged view. (c) Optical images of SHARK hydrogels. Scale bar = 5 mm. i. SHARK containing different concentrations of PCG (0, 0.8, 1.5, 3.0 and 4.5 mg mL^−1^). ii. Flat (top) and twisted (bottom) SHARK bands. iii. SHARK band held by tweezers. iv. SHARK balloon inflated with argon gas. (d) SHARK hydrogel (CPCG = 4.5 mg mL^−1^) stretched to ∼77 times its initial length in a tensile test. Scale bar = 5 mm. (e) Uniaxial tensile stress-strain curves of SHARK containing different concentrations of PCG (0, 1.5, 3.0 and 4.5 mg mL^−1^). (f) Uniaxial tensile stress-strain curves of SHARK (CPCG = 4.5 mg mL^−1^) under different deformation rates ranging from 5 to 30 mm min^−1^ (2 to 12 mm mm^−1^ min^−1^). Both the Young's modulus and the fracture strain depend on the deformation rate, revealing nonequilibrium disruption of weak interactions upon stretching. (g) Deformation rate dependence of the Young's modulus and fracture strain of SHARK in f.

We next prepared SHARK by direct photoinitiation of free radical aqueous polymerization of acrylamide in the presence of varying amounts of PCG sheets (Fig. 3a). As shown by scanning electrical microscopy (SEM) images, SHARK exhibited a typical interconnected porous hydrogel network (Fig. 3b, top). The POG sheets cross-linked with each other through the polymer and peptide network (Fig. 3b, bottom) perfectly matched the structures illustrated in Fig. 3a, indicating successful preparation of SHARK with designed microstructures. The water content was higher than 70% (Fig. S10a). In a typical reaction mixture, the concentration of acrylamide was fixed at 22.5% (w/w), and the concentration of the photoinitiator lithium phenyl-2,4,6-trimethylbenzoylphosphinate (LAP) was 0.1% (w/w). The solution was degassed, and the reaction proceeded for ∼0.5 h under UV illumination (285 nm and 253 mW cm^−2^) at room temperature. Stable hydrogels were formed, and the color darkened with increasing PCG content (Fig. 3c (i)). The hydrogel was elastic and could be twisted into a spiral shape or bent multiple times without causing permanent damage (Fig. 3c (ii) and (iii)). It could even be blown up like a balloon (Fig. 3c (iv) and movie S1 in the supplementary materials online). All these results demonstrated the excellent ductility of SHARK through the synergistic effects of the dynamic peptide self-assembly and layer shear of graphene (Fig. 3a). Moreover, SHARK could be stretched up to 77 times its original length without rupture (Fig. 3d and movie S2). The stretchability of SHARK outperformed that of many well-known ultrastretchable hydrogels, including the double network hydrogel [35,36], slide-ring hydrogel [37] and pseudopolyrotaxane hydrogel [38].

### Mechanical properties of SHARK

The mechanical properties of SHARK were quantitatively measured by standard mechanical tensile tests. Typical tensile stress-strain curves are shown in Fig. 3e, and a summary of the mechanical properties is listed in Table S2. The fracture strain and toughness of SHARK increased sharply with increasing PCG concentration. The fracture strain of SHARK containing 4.5 mg mL^−1^ PCG was 7736%, ∼13 times the strain of pure polyacrylamide hydrogel. The toughness of SHARK was 32.64 MJ m^−3^ (Table S3). Note that the Young's modulus and fracture strain depended on the deformation rates, indicating that the rupture of peptide-graphene interactions in hydrogels is a nonequilibrium process (Fig. 3f). Increasing deformation rates led to an increase in the Young's modulus and a decrease in the fracture strain (Fig. 3g). The hydrogel was crack-insensitive (Fig. S11a and movie S3). The fracture energy [35], a direct measure of the resistance to crack propagation, reached 19.75 kJ m^−2^ (Fig. S11b–d and Table S3). The hydrogel can maintain its reversibility in the range of ∼0–500% and showed larger hysteresis at higher strains in the stretching-relaxation curves, indicating that the interactions between Py and graphene can be ruptured to dissipate energy and prevent crack propagation (Fig. S12).

### Capacitive mechanical sensor based on SHARK

**Figure 4.**
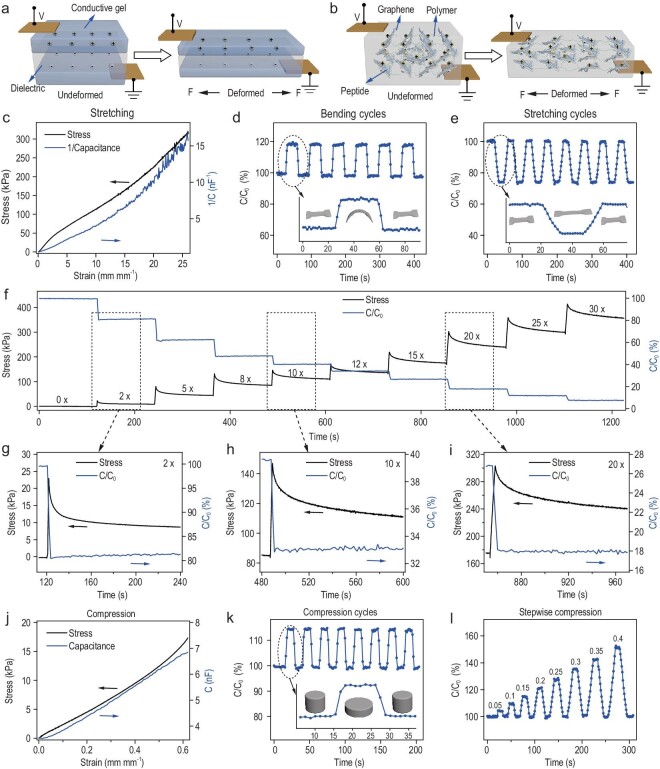
Capacitive mechanical sensor based on SHARK. (a and b) Comparison of the structure and sensing mechanism of sandwich-shaped artificial skin and SHARK. In sandwich-shaped artificial skin (a), the capacitance increased upon stretching because of deformation of the dielectric layer. In contrast, in SHARK (b), the capacitance decreased because of separation of graphene layers. (c) Reciprocal of capacitance (1/capacitance) vs strain and stress vs strain for a band-shaped SHARK sensor in the tensile strain range of 0–26 mm mm^−1^. (d and e) Change in the capacitance of a SHARK sensor in multiple bending (d) and stretching (e) cycles at a strain of 0.5 mm mm^−1^. Insets correspond to zoomed-in views of typical cycles. (f) Stress and capacitance relaxation of a band-shaped SHARK sensor at strains from 0 to 30 mm mm^−1^. (g–i) Zoomed-in views of stress and capacitance relaxation at strains of 2 (g), 10 (h) and 20 (i) mm mm^−1^. (j) Capacitance vs strain and stress vs strain curves for a disc-shaped SHARK sensor in the compressive strain range of 0–0.65 mm mm^−1^. (k and l) Change in the capacitance of a SHARK sensor in multiple compression cycles in the strain range of 0–0.15 mm mm^−1^ (k) and upon stepwise ramping at strains from 0 to 0.4 mm mm^−1^ (l). The strain refers to the normalized distance change of the two ends of the hydrogels. The inset in (k) corresponds to a zoomed-in view of a typical cycle.

We next explored strain and pressure sensing using SHARK. SHARK can be considered a bulk capacitor made of interconnected parallel microcapacitors (Fig. 1b). In a typical three-layer sandwich-shaped capacitive mechanical sensor, the change in capacitance results from deformation of the dielectric elastomer layer (Fig. 4a). When it is stretched to a deformation ratio of λ (deformed length/initial length), both the width and thickness of the elastomer shrink by a factor of λ, and the capacitance increases by a factor of λ. In contrast, the change in the capacitance of SHARK results from separation of the PCG electric layer upon deformation (Fig. 4b). Considering the hydrogel as soft insulator, the microcapacitors are formed with the conductive graphene sheets and insulative hydrogel between the graphene sheets (Fig. S13a). Upon compression, the distance of the PCG decreased, leading to an increase in capacitance (Fig. S13b). Upon stretching, the separation of the PCG layers increased, leading to a decrease in capacitance (Fig. S13c). When it is stretched to a deformation ratio, both the width and thickness of the hydrogel shrink by a factor of (deformation ratio)^−0.5^, and the capacitance increases by a factor of deformation ratio. The observed strain-dependent capacitance verified this mechanism (Fig. 4c). The reciprocal of capacitance increased by more than 35 times when the strain increased to 2600%, linearly related to the strain and stress at the same time. Moreover, the frequency response of the dielectric loss (tan δ) is tuneable by the applied strain (Fig. S14). The characteristic response frequency shifted to lower values at higher strains, presumably because of decreased turnover frequency of the ‘dipole’ (graphene nanosheet pairs) brought about by the increased space between PCG nanosheets. Note that capacitance hysteresis was not obvious because the capacitance is only sensitive to the separation between graphene sheets and not to the physical interactions bonding these sheets (Fig. S15). Therefore, SHARK was able to provide reliable tensile strain sensing up to ∼2600% strain with a sensitivity (gauge factor calculated based on 1/C) of 1.39. To test the reversibility and stability of the strain sensing, SHARK was subjected to cyclic bending and stretching (Fig. S16a and b). The capacitance increased by ∼20% in bending cycles and decreased by ∼27% in stretching cycles when the strain changed from 0 to 0.5 mm mm^−1^ (Fig. 4d and e). The zoomed-in plot shows that the response times of the sensor were in the range of a few seconds (insets of Fig. 4d and e). The capacitance variation remained consistent over more than 1000 bending cycles and 5000 stretching cycles (Fig. S17) for polydimethylsiloxane (PDMS)-coated band-shaped SHARK, indicating the great stability and anti-fatigue properties of the strain sensor. Particularly, the stress-relaxation experiments at various strains indicated that although the stress gradually decreased with relaxation time, the capacitance was almost constant throughout (Fig. 4f–i), further confirming the outstanding ability for strain sensing.

Next, we explored pressure sensing using SHARK by measuring change in capacitance at various pressures (Fig. 4 j–l and S16c). The pressure sensitivity, measured by the slope of the capacitance-pressure trace, was almost constant in the range of 0–20 kPa, with a sensitivity of 0.2 nF kPa^−1^ and a gauge factor of 1.49 (Fig. 4j). Cyclic and stepwise pressure tests indicated that the sensor could quickly respond to changes in pressure and returned to its original state when the pressure was released (Fig. 4k and l). The PDMS-coated disc-shaped pressure sensor did not show any obvious fatigue in its pressure response even after more than 2000 compression-relaxation cycles, indicating its great stability and reversibility in pressure sensing (Fig. S17c).

### Complex motion sensing using SHARK

**Figure 5.**
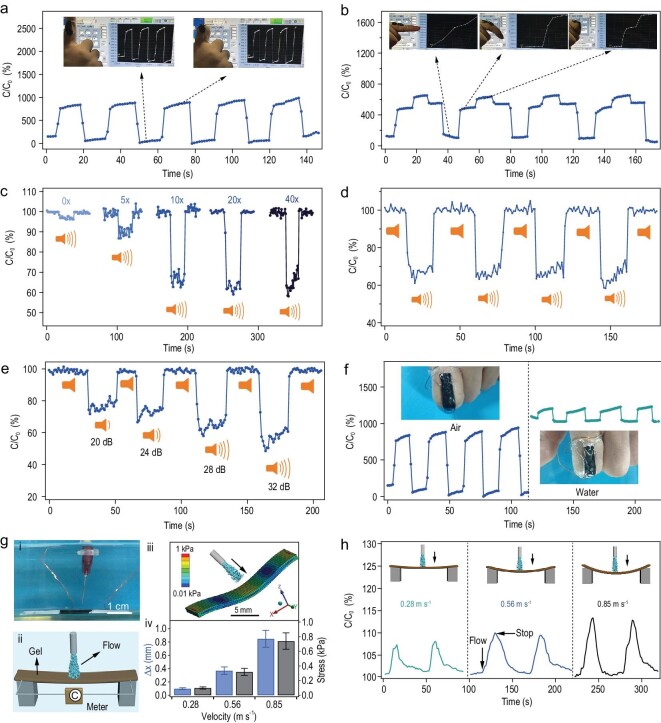
Complex motion sensing using SHARK in both air and aqueous environments. (a and b) Relative capacitance change of band-shaped SHARK fixed on a human finger upon bending in two (a) or three (b) states. Insets show the movements of the finger bearing the sensor. (c) Change in the capacitance of SHARK when exposed to sound (Music ‘Go time’, Mark Petrie, USA, ∼32 dB) at various pre-strains (λ = 0, 5, 10, 20 and 40 mm mm^−1^). (d) Change in capacitance upon reversibly turning the music on and off at a pre-strain of 40 mm mm^−1^. (e) Response of SHARK to different sound intensities (pre-strain: λ = 40 mm mm^−1^). (f) Comparison of the motion sensing using the same sensor in air (left) and in water (right). Insets present the finger bearing the SHARK sensor. (g) Sensing flow rates using SHARK in water. (i) Image of the experimental setup. (ii) Electric circuit. (iii) Stress nephogram of the sensor at a flow rate of 0.85 m s^−1^ simulated by ANSYS. (iv) Summary of the simulated largest deformation (Δx, blue) and stress (black) in the z-axis direction. (h) Change in the capacitance of the sensor in response to flows of various rates (0.28, 0.56 and 0.85 m s^−1^) in water.

We further explored the application of SHARK for complex motion sensing. We attached SHARK to a forefinger by flexible tape to monitor finger motions (Fig. 5a and b). The capacitance of the device was enhanced by ∼800% with complete bending and returned to its original level after holding the finger straight (Fig. 5a). The response amplitude of the devices was much higher than those of devices constructed using the sandwich configuration, which were usually less than 100% [39–41]. The deformation of the device during the finger bending motion results from coupled bending, compressing, and stretching motions, among which bending and compressing contribute the most. All these motions contribute to the overall capacitance increase. The response amplitudes of the device decreased at reduced thickness and still remained more than 300% even at a thickness of 0.2 mm (Fig. S18). Moreover, the sensor could precisely respond to different levels of bending and provide reliable measurements for multiple cycles (Fig. 5b and movie S4).

Having demonstrated the exciting sensing ability for large-scale motions, we then explored the sensing of high-frequency and low-amplitude sound. We found that the sensitivity of SHARK to acoustic waves was significantly enhanced by a pre-strain (Fig. 5c), probably because of the pre-strain leading to alignment of PCGs in SHARK along the stretching force [42]. Such a structural rearrangement shifted the resonance frequency to lower values, making it more sensitive to sound (Fig. S14). The change in capacitance increased with increasing pre-strain (λ = 0, 5, 10, 20 and 40 mm mm^−1^) when exposed to an acoustic wave (music: Go time, Mark Petrie, USA) of ∼32 dB (Fig. 5c). At a λ of 40, the capacitance decreased quickly by ∼40% when the music was turned on and recovered when the music was stopped (Fig. 5d and movie S5). The change in capacitance was reversible, and the amplitude of the change directly correlated with the volume of the sound (20, 24, 28 and 32 dB) (Fig. 5e and movie S6).

Furthermore, the large capacitive change and high sensitivity allowed the device to function well in environments with high background electrical noise, such as aqueous environments. Additional bisacrylamide (0.5% w/v) was added during the preparation of SHARK used in water to enhance the erosion resistance (Fig. S19). The sensor can fiducially resolve the same stretching, compression and finger bending motions in water despite the maximal capacitive variation percentage decreasing (Fig. S20 and 5f). Moreover, SHARK was designed to directly sense microflows in water (Fig. 5g (i) and (ii)). In the proof-of-principle demonstration, we applied flow perpendicular to the device surface to avoid complications from the geometry of the fluid chamber to the actual flow velocity. The sensor would deform to various degrees in response to flows of different velocities according to the simulation using ANSYS, thus sensing flow velocity and stress in water (Fig. 5g (iii) and (iv)). As expected, capacitive enhancements of different amplitudes were observed with scale-up of the flow velocity (0.32, 0.56 and 0.85 m s^−1^), fully consistent with the analogue simulation results (Fig. 5h).

### Self-healing and remolding of SHARK

**Figure 6.**
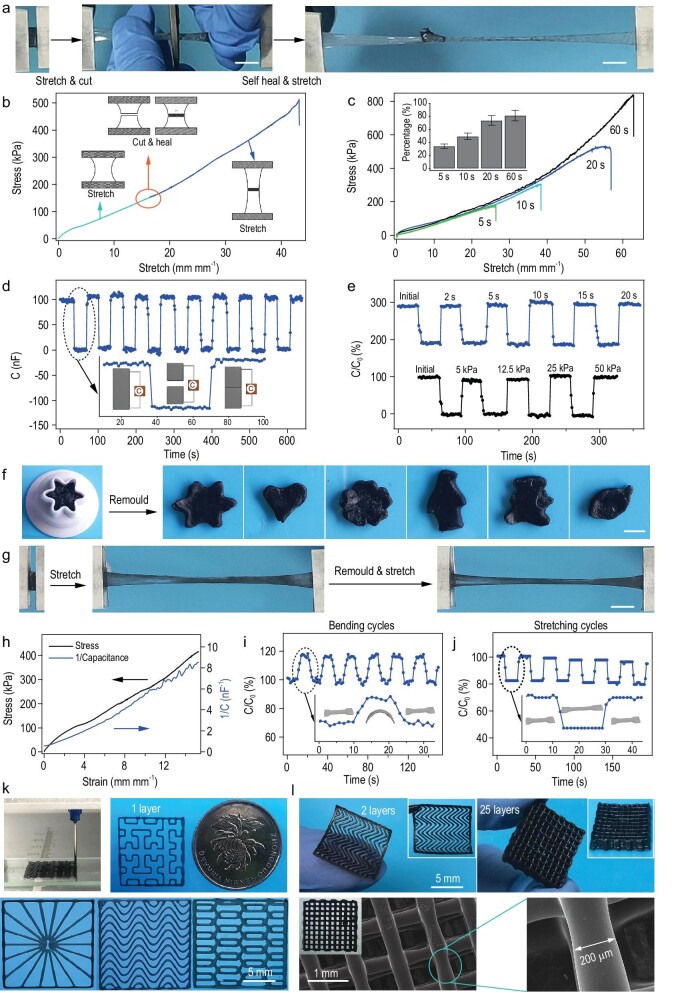
Self-healing and remolding of SHARK. (a) Optical images showing cutting, rejoining and stretching of SHARK. The band was only knotted to allow healing for seconds under stress. Scale bar = 5 mm. (b) Stress-strain curves of the initial and healed SHARK band in a continuous test. Insets correspond to schematics of the original, cut and healed samples. (c) Typical stress-strain curves of hydrogels healed for different healing times at room temperature (healing pressure ∼25 kPa). The inset shows the time-dependent recovery percentage of fracture strain. (d) Time evolution of multiple electrical healing processes for the same sample by gently touching the broken surfaces together (healing pressure ∼25 kPa, healing time ∼5 s). (e) Healing of the electrical properties after different healing times (top, healing pressure ∼25 kPa) and pressures (bottom, healing time ∼5 s). (f) Remolding of SHARK into different shapes. Scale bar = 5 mm. (g) Stretching the same sample before and after remolding. Scale bar = 5 mm. (h) Reciprocal of capacitance (1/capacitance) vs strain and stress vs strain for a remolded band-shaped SHARK sensor in a tensile strain range of 0–15 mm mm^−1^. (i and j) Change in the capacitance of remolded SHARK in multiple bending (i) and stretching (j) cycles at a strain of 0.5 mm mm^−1^. Insets show schematics of the bending and stretching cycles. (k) Demonstration of extrusion-based 3D printing of SHARK into different 2D shapes. (l) Optical and SEM images of multiple-layer mesh structured SHARK prepared by 3D printing.

Because of its single-layer structures, the mechanical and electrical properties of SHARK-based sensors were fully self-healable. As illustrated in Fig. 6a and Movie S7, if a stretched SHARK band was cut into two pieces during stretching and then quickly pressed together at room temperature, the cut pieces could merge together within several seconds. Moreover, the merged hydrogel could still be stretched to more than 40 times its original length without rupture. The stress-strain curves of the hydrogel before and after self-healing are shown in Fig. 6b. The strain-stress curves before and after healing merged together, indicating that the elasticity was almost fully restored. The recovery of the maximal strain depended on the healing time (Fig. 6c). A SHARK hydrogel was self-healable even at a strain of λ = 73 mm mm^−1^ and could be plucked like an elastic rubber string (Movie S8), illustrating the great mechanical self-healing properties of SHARK.

Figure 6d and e shows the time evolution of multiple electrical healing processes for the same device. The capacitance dropped to almost zero when SHARK was cut. However, when the two surfaces were brought into contact and gently pressed, the capacitance returned to almost the original value in less than 5 s, and no obvious change in the recovered capacitance was observed even after the device was cut and healed 10 times (Fig. 6d). Although a longer compression time or a higher compression stress could improve the healing of the mechanical properties, it did not greatly affect the capacitance recovery (Fig. 6e). Such an outstanding electrical self-healing property can be attributed to the unique structure of SHARK. Upon cutting, the majority of the PCG electric double layers are not affected. Moreover, the capacitance can be almost fully restored once the broken surfaces barely touch each other without any compression (Fig. S21 and Movie S9). We define the capacitive healing efficiency η_ele_ as the proportion of capacitance recovered with respect to the initial value of the pristine sample before cutting. η_ele_ remained as high as 90% when successively dividing and rejoining the devices at the same location 10 times, demonstrating the great electrical self-healing properties of SHARK. Furthermore, the sensing capability of healed band-shaped SHARK was also fully restored after cutting/healing cycles (Fig. S22).

More importantly, SHARK was remoldable because the interactions between the peptide and graphene are non-specific and reversible (Fig. 6f and g). The remolded hydrogels resembled the original hydrogels in terms of both mechanical and electrical properties (Fig. 6h), as well as the performance in cyclic sensing tests (Fig. 6i and j). The maximal strain decreased after remolding but was still greater than 3500% after four fracture-remolding cycles (Fig. S23). The remolding and strain/shear thinning properties of SHARK (Fig. S24) make it printable for the construction of flexible iontronics. Figure 6k and movie S10 show the typical 3D extrusion printing process and various single-layer complicated structures based on SHARK. The size of the printed iontronic device could be as small as a one-yuan coin. Multilayer flexible pressure sensor chips could also be fabricated, and the microstructures were investigated (Fig. 6l). The layered structures could be self-standing and were ordered, in which the smooth printed fibers exhibited a uniform diameter of ∼200 μm.

## DISCUSSION

In this work, we have demonstrated successful design of single-layer hydrogel artificial skin, or ‘SHARK’. In contrast to the sandwich structure of the widely used hydrogel-based capacitive artificial skin, SHARK contains only a single hydrogel layer with interconnected microcapacitors formed by dielectric molecule-coated conductive sheets (i.e. graphene). Unlike typical three-layer sandwich-shaped capacitive mechanical sensors, in which the overall capacitance is proportional to the surface area of the hydrogel layer and reversely proportional to the thickness of the elastomer layer, the capacitance of ‘SHARK’ is determined by the spatial distribution of the graphene sheets in the hydrogel network. Any minute structural changes to the overall 3D hydrogel network can lead to a drastic change in capacitance over the two electrodes. Moreover, the coated peptide fibers not only increase the dielectric constant but also increase the water solubility and prevent restacking of the graphene layers. Such a design greatly improves the sensitivity and allows complete self-healing of mechanical and electrical properties after damage. The strain sensing range, sensitivity and self-healing ability of SHARK outperform most of the other capacitive strain sensors reported so far in the literature (Table S4).

Although graphene/graphene oxide-peptide based hydrogels have been reported in quite a few cases [43–46], the reported hydrogels did not exhibit combined high stretchability and fast healing. Generally, to make the hydrogel stretchable, the crosslinks should be strong enough to withstand considerable forces. In SHARK, the interfacial interactions between peptide and graphene are rationally tuned through interfacial self-assembly. Individual pyrene-graphene interactions are dynamic and ruptured at low forces. However, upon self-assembling on graphene surface, pulling off a peptide simultaneously ruptures interactions with graphene and interactions with neighboring peptides. Therefore, the rupture forces are considerably enhanced, as confirmed by our single molecule AFM experiments. Moreover, the polymer network forms multiple bonds with each graphene sheet. This can considerably decrease the forces loaded on individual peptide-graphene bonds and makes complete detachment of multiple bonds difficult [47]. Besides, SHARK shows a similar necking phenomenon to double network hydrogels, further enhancing the stretchability and crack-insensitive property [48]. Therefore, SHARK can tolerate local defects and be stretched to more than 77 times its original length. On the other hand, in such multiple bonding scenarios, the detached bonds can rebind quickly as they are constrained in space by other undetached bonds. Thanks to strong, dynamic and fast-recovery interfacial interactions between the self-assembled peptides and graphene, SHARK is also self-healing, remoldable and printable. These unique traits will greatly facilitate construction of mechanical sensors of complicated shapes and structures, as well as integration of such sensors with other functional units.

The mechanical sensing mechanism of SHARK is based on a change in capacitance, which is also distinct from other graphene-containing hydrogel sensors that are based on a change in conductivity. As all graphene sheets in SHARK are mostly covered by dielectric self-assembled peptide fibers, the graphene sheets are well separated. Therefore, the conductance of SHARK is contributed by the mobile ions in hydrogels rather than conductive graphene layers. The conductance (<0.01 S m^−1^) does not change too much upon stretching and is more than one order of magnitude lower than that of graphene-based conductive hydrogels [49,50]. Because the capacitance of SHARK does not depend on the connectivity of graphene sheets, the sensor is able to measure large strains. The pre-strained hydrogels show even better response to high-frequency and low-amplitude sound than unstrained ones. In contrast, the conductive graphene-based hydrogel sensors cannot sense large strains because the sensitivity is sharply decreased at large strains due to the disconnection of the conductive graphene sheets.

It is worth mentioning that the performance of hydrogel-based mechanical sensors can be affected by water content. We found that water evaporation in SHARK is much slower than in regular polyacrylamide hydrogels, presumably because the graphene layers are water-impermeable and can prevent migration of water to the hydrogel surface (Fig. S10b). Despite water loss changing the capacitance value of the device, the ratio of the capacitance change (ΔC/C0) does not vary too much. Moreover, unlike other physical hydrogels, SHARK erodes very slowly in water because of the strong interfacial bonding between peptide and graphene. These properties ensure short-term use of SHARK for mechanical sensing. To use the sensor for long-term applications, the stability of the hydrogels can be further improved by laminating a surface-bonded elastomer layer, which will be our next endeavor.

## CONCLUSION

In summary, in this work we demonstrated the design and engineering of a single-layer hydrogel artificial skin that is strong, tough and able to fully self-heal its mechanical and electrical properties after damage. We illustrated successful applications of the hydrogels as strain and pressure sensors for complicated motion monitoring, sound sensing and flow detection in air or water. The great processability of SHARK in both bulk remolding and 3D printing allows construction of designer self-healable sensor chips. With the improved mechanical, electrical and self-healing properties, we expect this novel capacitive hydrogel sensor to have broad applications in next-generation flexible iontronics.

## METHODS

### Preparation of SHARK

To obtain the PCG dispersion for hydrogel preparation, Py-GAGAGY-ACLT instead of Py-GAGAGY was used in exfoliation of graphene so that the peptides on the graphene sheets could integrate with the polyacrylamides via copolymerization. Then, the obtained PCG dispersions were concentrated to a PCG concentration of 6 mg mL^−1^ with a nitrogen dryer. In a typical preparation of hydrogels, acrylamide was dissolved in a PCG dispersion to a concentration of 225 mg mL^−1^. The mixture was degassed for 30 min by blowing argon. LAP was added to a concentration of 1.0 mg mL^−1^ as a photoinitiator for acrylamide and Py-GAGAGYK-ACLT. The mixture was placed under ultraviolet light (285 nm, 253 mw cm^−2^) for 0.5 h to finish the copolymerization. The temperature increase brought by the heat effects was ∼3–4°C and did not lead to degradation of the peptide. Because the peptide self-assembly is achieved before the addition and copolymerization of acrylamide, the β-sheet structures of the peptide is still kept in the presence of (poly)acrylamide. The resulting hydrogel was stored under seal at room temperature before the mechanical tests. For hydrogels used in aqueous measurements, additional bisacrylamide (0.5% w/v) was added to improve the stability of hydrogels in water.

### Tensile test

Tensile stress-strain measurements were performed using a tensile-compressive tester (Instron-5944 with a 10 N sensor) in air at room temperature. Unless otherwise noted, the strain rate of stretching was maintained at ∼10 mm min^−1^ (4.0 mm mm^−1^ min^−1^). The strain, λ, was defined by the distance displacement between the two clamps when the gel was deformed, divided by the distance when the gel was undeformed. The Young's modulus corresponded to the approximate linear fitting value under 1 mm mm^−1^ strain deformation. The toughness was calculated from the area below the tensile stress-strain curve until fracture. For the healing experiments, SHARK bands were cut into pieces and pressed after bringing the cut pieces back into contact at room temperature in air.

### Electrical tests and motion sensing

All electrical tests were performed with an LCR meter (HIOKI-IM3536, Japan) in air at a voltage of 1 V and a frequency of 100 000 Hz unless otherwise stated. The data were collected with a custom-written LabView script. The capacitance vs strain was recorded by coupling the LCR meter with the tensile/compressive tester (Instron-5944, USA). For the motion sensing at different modes, the capacitance vs different stimulations (motions, environments, frequency and acoustic waves) were recorded and analyzed with Igor 6.0 (Wavemetrics, Inc.).

## Supplementary Material

nwab147_Supplemental_Files
