# Supplementary material for: Stretchable and self-healable hydrogel artificial skin
Source: Natl Sci Rev. 2021 Aug 14;9(7):nwab147. doi: 10.1093/nsr/nwab147 (PMC9375542; doi:10.1093/nsr/nwab147)
Supplement: nwab147_Supplemental_Files [file nwab147_supplemental_files.zip › Supplementary_Materials.docx]

**Supplementary Materials for**

**Stretchable and self-healable hydrogel artificial skin**

Bin Xue^1#^, Hui Sheng^1#^, Yongqiang Li^1^, Lan Li^2^, Weishuai Di^1^, Zhengyu Xu^1^, Linjie Ma^1^, Xin Wang^1^, Haoting Jiang^1^, Meng Qin^1^, Zhibo Yan^1^, Qing Jiang^2^, Jun-Ming Liu^1^, Wei Wang^1,3^*, Yi Cao^1,3,4^ *

*^1^ National Laboratory of Solid State Microstructures, Department of Physics, Nanjing University, Nanjing, 210093, China.*

*^2^ State Key Laboratory of Pharmaceutical Biotechnology, Department of Sports Medicine and Adult Reconstructive Surgery, Drum Tower Hospital affiliated to Medical School of Nanjing University, Nanjing, 210008, China.*

*^3^ Institute for Brain Sciences, Nanjing University, Nanjing, 210093, China.*

*^4^ Chemistry and Biomedicine innovation center, Nanjing University, Nanjing, China 210093.*

* **Correspondence to:** caoyi@nju.edu.cn, wangwei@nju.edu.cn.

^#^ Equally contributed to this work

**This file includes:**

Supplementary Experimental Section

Figure S1 to S24

Tables S1 to S4

Captions for Movie S1 to S10

**Other Supplementary Information for this manuscript include the following:**

Movie S1 to S10

**Experimental Section**

**Materials**

Graphite powder (~200 mesh) and lithium phenyl-2,4,6-trimethylbenzoylphosphinate (LAP) were purchased from Alfa Aesar (Thermo Fisher Scientific Inc., US). HOPG (highly oriented pyrolytic graphite) was purchased from the Institute of Metal Research, Chinese Academy of Sciences (China). Mica was purchased from Zhongjingkeyi Technology Co., Ltd. (China). Acrylamide was purchased from Sigma-Aldrich (Shanghai, China). The designed peptides were purchased from GL Biochem Ltd. (Shanghai, China). NHS-PEG-thiol (MW: 5 kDa), NHS-PEG-CH_3_ (MW: 2 kDa) and NHS-PEG**-**maleimide (MW: 5 kDa) were purchased from Nanocs, Inc. (USA). NH_2_-PEG-CH_3_ (MW: 10 kDa) was purchased from Laysan Nio, Inc. (USA). All other chemical reagents, unless otherwise stated, were purchased from Sinopharm Chemical Reagent Co., Ltd. (China). Milli-Q water was produced by a Milli-Q® integral water purification system (Merck KGaA, Germany). All reagents were used without further purification.

**High-performance liquid chromatography (HPLC) and MALDI-TOF analysis**

The designed peptides were synthesized by the solid-phase-based method and purified by HPLC. HPLC analysis was performed on a Thermo Scientific U3000 system with a 250×4.6 mm Fortis Xi 5 μm C18 column. For the Py-GAGAGY peptide, the mobile phases were solvent A, 0.1% TFA in H_2_O, and solvent B, 0.1% TFA in CH_3_CN, with the following gradient: 0 to 25 min, 66% to 41% A; 25.1 to 30 min, 41% to 0% A. For the Py-GAGAGYK-ACLT peptide, the mobile phases were solvent A, 0.1% TFA in H_2_O, and solvent B, 0.1% TFA in CH_3_CN, with the following gradient: 0 to 25 min, 35% to 60% A; 25.1 to 30 min, 60% to 100% A. All the samples were dissolved in methanol. The flow rate was 1.0 mL min^-1^, and the monitoring wavelength was 220 nm.

MALDI-TOF: Py-GAGAGY [C_37_H_37_N_5_O_8_] calcd. 679.26, found: 679.11.

Py-GAGAGYK-ACLT [C_46_H_51_N_7_O_10_] calcd. 861.37, found: 861.79.

**Preparation of thiol-coated cantilevers and peptide-coated HOPG substrates**

Standard silicon nitride (Si_3_N_4_) cantilevers were obtained from Bruker (type: MLCT). The cantilevers were immersed in chromic acid for 20 min at 95 °C and cleaned with Milli-Q water. Then, the cantilevers were immersed in 1% (v/v) (3-aminopropyl) triethoxysilane (APTES) dimethylsulfoxide (DMSO) solutions for 1 h to introduce amino groups to the surface, followed by rinsing with DMSO and Milli-Q water. After drying under nitrogen, cantilevers were immersed in a DMSO solution containing 1 mg mL^-1^ NHS-PEG-thiol (5 kDa) and 2 mg mL^-1^ NHS-PEG-CH_3_ (2 kDa) for another 1.5 h. NHS-PEG-CH_3_ was introduced to cover the unreacted amino groups. Finally, the thiol-coated cantilevers were washed with DMSO and Milli-Q water before being dried with nitrogen.

For the preparation of the peptide- or 1-pyrenecarboxylic acid-coated HOPG substrates, the HOPG surface was freshly cleaved before use. Then, the HOPG substrates were immersed in a DMSO solution containing 0.5 mg mL^-1^ Py-GAGAGY peptide or 0.17 mg mL^-1^ 1-pyrenecarboxylic acid for 1 h. Then, the substrates were rinsed with DMSO and Milli-Q water, dried under nitrogen and immersed in DMSO solutions containing 2 mg mL^-1^ NHS-PEG-maleimide (5 kDa) for 1.5 h to introduce the maleimide group. After being washed with DMSO and Milli-Q water again, the HOPG substrates were dried with nitrogen. All the cantilevers and substrates were used immediately after the preparation was completed.

**Single-molecule atomic force microscopy (AFM) experiments**

Single-molecule force spectroscopy (SMFS) experiments were carried out on a commercial AFM system (JPK ForceRobot 300) in an aqueous solution containing 0.05% (v/v) polyoxyethylene sorbitan monolaurate (Tween 20) at room temperature (~25 °C). Thiol-coated cantilevers (spring constant of ~0.05 N m^-1^) were used in all experiments. The spring constant was calibrated using the equipartition theorem each time. In a typical SMFS experiment, the cantilever was brought into contact with the surface of the substrate at a contact force of ~300 pN for 500 ms to trigger the reaction of thiol and maleimide. Then, the cantilever was pulled back to obtain the force-extension curves. All force curves were collected by commercial software from JPK and analysed offline using a custom-written protocol in Igor 6.0 (Wavemetrics, Inc.).

**Dynamic force spectroscopy experiments**

The dynamic force spectroscopy experiments were performed using different pulling speeds (200, 400, 800, 1600 and 3200 nm s^-1^). The dissociation rate (*k_off_*) and potential width (*Δx*) could be obtained by fitting the data using the Bell-Evans equation (1, 2).

$F=\frac{k_{B}T}{\Delta x}\ln\left( \frac{\Delta x}{k_{off}k_{B}T} \right) +\frac{k_{B}T}{\Delta x}\ln\left( r \right)$ (1)

*F* is the most probable rupture force, *k_B_* is the Boltzmann constant, *T* is the absolute temperature and *r* is the loading rate.

**Synthesis of Py-GAGAGY-mPEG**

The mPEG-connected Py-GAGAGY (Py-GAGAGY-mPEG) was prepared by connecting CH_3_-PEG-NH_2_ (10 kDa) to the C-terminus of the Py-GAGAGY peptide in the presence of O-benzotriazole-N, N, N', N'-tetramethyl-uronium-hexafluorophosphate (HBTU) and N, N-diisopropylethylamine (DIPEA). Typically, the mPEG and Py-GAGAGY peptide were dissolved in DMSO to concentrations of 0.5 and 4.0 mM, respectively. Then, HBTU and DIPEA were added into the solution to a concentration of 4.0 mM. The mixture was stirred for 24 h at room temperature. Finally, the unreacted reactants were removed by dialysis in Milli-Q water, and the product was lyophilized. The linking efficiency of the resulting Py-GAGAGY-mPEG was confirmed with UV calibration curves (Figure S5).

**Fourier transformed infrared (FT-IR) spectroscopy measurements**

FT-IR spectra was recorded with a NICOLET iS10 (NICOLET, USA) spectrometer. The peptide was dispersed into dimethyl sulfoxide-d6 and then diluted into D2O to a final concentration of 5 mg mL^-1^. Then, the samples were stored at room temperature (25 °C) for at least 2 h, and the FT-IR spectra were measured with the liquid samples directly. The reported spectra were the average of more than 20 scans with the background signals subtracted. For the peptide co-assemblies, Py-GAGAGY and Py-GAGAGY-mPEG or Py-GAGAGYK-ACLT and Py-GAGAGY-mPEG were mixed at a molar ratio of 10:1. Then, the FT-IR spectra were recorded with the same method described above.

**Production of peptide-coated graphene (PCG) via aqueous exfoliation**

In a typical graphene exfoliation process, powders of bulk graphite and Py-GAGAGY-mPEG were suspended in Milli-Q water to concentrations of 3 and 1.5 mg mL^-1^, respectively. Py-GAGAGY was first dissolved in DMSO to a concentration of 100 mg mL^-1^ and diluted to 1.2 mg mL^-1^ using Milli-Q water. Then, the three kinds of solutions were mixed with equal volumes, and the final concentrations of graphite, Py-GAGAGY-mPEG and Py-GAGAGY were 1, 0.5 and 0.4 mg mL^-1^, respectively. The as-prepared solutions (10 mL in a 15 mL centrifuge tube, Corning) were treated with a medium power tip sonicator (XO-1000D, Nanjing Xian’ou Instruments Manufacture Co., Ltd, China) operating at 20 kHz in an ice bath. The diameter of the ultrasonication tip was 6 mm, and the density of the ultrasonication power was 14.6 W cm^-2^. The sonication was switched on and off every 3 s with a total time of 3 h for all samples. All the products were stored at 4 °C for 3 days and then centrifuged at 8000 rpm for 10 min at room temperature. For the exfoliation of graphene with different molar ratios of Py-GAGAGY-mPEG and Py-GAGAGY (1:5, 1:10, 1:15 and 1:20), stock solutions containing different ratios of Py-GAGAGY-mPEG and Py-GAGAGY were prepared, and graphene was obtained as described above.

**Estimation of the concentrations of graphene**

The concentrations of the graphene solutions were estimated by UV-Vis absorption spectra (Jasco V550, Japan), and 1390 mL mg^-1^ m^-1^ was used as the absorption coefficient of graphene at 660 nm (3).

**Transmission electron microscopy (TEM)**

TEM was performed using a Tecnai F20. The PCG solution was dialysed against Milli-Q water at 4 °C to remove the absorbed peptides. A drop of the obtained graphene dispersion was scattered on a carbon film-coated grid (230 mesh) and dried at room temperature for the TEM measurement.

**AFM imaging**

The AFM samples were prepared as follows: The as-prepared PCG dispersion was diluted to a concentration of 0.1 mg mL^-1^. Then, 50 μL of the newly prepared solution was loaded onto a freshly peeled mica surface and allowed to adsorb for 5 min. Then, the majority of the solution was carefully removed, and the mica surface was allowed to dry at room temperature. The surface was further blown with compressed air to remove any loosely attached material prior to the AFM measurement. Then, the samples were imaged at room temperature by a NanoWizard ULTRA Speed (JPK, Germany) operating in tapping mode (conditions: scan rate, 1 Hz; pixel number, 1024 × 1024). Silica cantilevers (OLTESPA, Bruker, USA) with typical tip radii of ~7 nm and resonance frequencies of ~96 to 175 kHz were used for imaging.

For the imaging of pyrenecarboxylic acid, self-assembled peptides and co-assembled peptides on HOPG, 50 μL of newly prepared pyrenecarboxylic acid (0.1 mg mL^-1^) or peptide solution (0.5 mg mL^-1^) was loaded onto a freshly peeled HOPG surface and allowed to adsorb for 5 min. After removing the majority of the solution and drying at room temperature, the samples were imaged with AFM in the same way as described above.

**X-ray photoelectron spectroscopy (XPS) measurements**

The graphene dispersion was dialysed in Milli-Q water to remove peptides. Then, the samples were freeze-dried to remove the solvent. XPS measurements of the powder samples were carried out with a PHI 5000 Versa Probe (UlVAC-PHI, Japan).

**Raman spectroscopy measurements**

In a typical measurement, a drop of PCG solution was dried on a silicon slice to form a graphene film and characterized with a confocal Raman microscope (alpha300, WITec) with a 532 nm laser under atmospheric conditions. The microscope was equipped with a piezo scanner (P-500, Physik Instrumente) and a 100× objective (Nikon, NA 0.6). The laser powers were less than 2 mW during the measurements. The Raman scattered light was detected by a thermoelectrically cooled CCD detector (DU401A-BV, Andor) with an integration time of 10 s and 3 accumulations. The measurement and subsequent data analysis were performed with the software ScanCtrlSpectroscopyPlus (Version 1.38, WITec) and Project FOUR (Version 4.1 WITec).

The Raman spectra of the prepared graphene showed similar features in terms of the G (~ 1560 cm^-1^) and D (~ 1360 cm^-1^) peaks. The ratio between the intensity of the D and G peaks varies inversely with the size of the nanosheets. The 2D peak in monolayer graphene is a single peak, whereas it splits into several bands in multiple-layer graphene. The number of layers (N_G_) and lateral size (L) of the graphene can be estimated with Raman metrics (4).

$N_{G}={10}^{0.84M+0.45M^{2}}$ (2)

$M=\left[ \frac{I_{Graphene}\left( \omega_{p} \right)}{I_{Graphene}\left( \omega_{p} \right)} \right]/\left[ \frac{I_{Graphite}\left( \omega_{s} \right)}{I_{Graphite}\left( \omega_{s} \right)} \right]$ (3)

$\frac{I_{Graphene}\left( D \right)}{I_{Graphene}\left( G \right)}=\frac{I_{Graphite}\left( D \right)}{I_{Graphite}\left( G \right)}+\frac{k}{<L>}$ (4)

in which $I_{Graphite}\left( \omega_{p} \right)$ and$I_{Graphite}\left( \omega_{s} \right)$ are the intensities of the graphite 2D peak and the peak shoulder at −30 cm^−1^ relative to the 2D peak, $I_{Graphene}\left( \omega_{p} \right)$ and $I_{Graphene}\left( \omega_{s} \right)$are the intensities of the graphene spectrum at the 2D peak and the 2D peak shoulder, $I\left( D \right)$ is the intensity of the D peak, $I\left( G \right)$ is the intensity of the G peak, and $k$ is the slope previously estimated (5, 6).

**Water content measurements**

The hydrogel samples were weighed, and the wet weight was recorded as W_1_. Then, the hydrogel samples were lyophilized, and the weight was recorded again as W_2_. The water content (*ʂ*) was calculated as *ʂ =(1-W_2_/W_1_) ×100%*.

**Pure shear test**

A pure shear test was also used to characterize the toughness, following the method established in the literature (7-10). Two different samples, notched and unnotched, were used to measure the fracture energy T. The samples were prepared into a rectangular shape with a width of 8.0 mm (a_0_) and a thickness of 1.0 mm (b_0_). An initial notch of 1.6 (20%) mm in length was cut using a razor blade. The test piece was clamped on two sides, and the distance between the two clamps was set at 2.5 mm (L_0_). The upper clamp was pulled upward at a constant velocity of 10 mm min^-1^ (4.0 mm mm^-1^ min^-1^), while the lower clamp was fixed. The force-length curves of the samples were recorded, and the fracture energy was calculated from T = U(L_c_)×L_0_, where U(L_c_) is the area below the tensile stress-strain curve of the unnotched sample at the critical stretching distance L_c_, and L_c_ is the distance between the two clamps when the crack starts to propagate in the notched sample. The onset of crack propagation was determined using the movie images recorded by a camera (Figure S11).

**Motion sensing at different modes**

For the bending cycles, the band-shaped SHARK sensor (length: 8.0 mm, width: 8.0 mm, thickness: 1 mm) was fixed on the tensile/compressive tester. Then, the band was bent until the distance of the two ends decreased by half and released and the bending radius was 2.6 mm (Figure S16a). For the stretching cycles, the fixed band-shaped SHARK sensor was stretched to a strain of 0.5 mm mm^-1^ and released (Figure S16b). For the multi-compression tests, the disc-shaped SHARK sensor (height: 6 mm, diameter: 6 mm) was compressed to a strain of 0.5 mm mm^-1^ and released continuously (Figure S16c). For the stepwise compression tests, the disc-shaped SHARK sensor was compressed to different strains and released.

For tensile sensing, the sensor’s sensitivity or gauge factor (GF) was calculated by $GF=\frac{({\frac{1}{C}}_{\varepsilon}-{\frac{1}{C}}_{\varepsilon0})/(\frac{1}{C_{\varepsilon0}})}{\varepsilon}$, in which 𝜀 is the strain of the sensor and C_ε0_ and C_ε_ are the original capacitance at ε = 0 and the capacitance at a strain, respectively. For compression sensing, the sensor’s sensitivity or GF was calculated by $GF=\frac{(C_{\varepsilon}-C_{\varepsilon0})/C_{\varepsilon0}}{\varepsilon}$, in which 𝜀 is the strain of the sensor and C_ε0_ and C_ε_ are the original capacitance at ε = 0 and the capacitance at a strain, respectively.

For the frequency response of the dielectric loss, the tan δ of SHARK in the alternating electric field frequency range of 100-1,000,000 Hz was recorded at different pre-strains. tan δ was defined as $tan\delta=\varepsilon^{''}/\varepsilon^{'}$, in which $\varepsilon^{''}$ and $\varepsilon^{'}$ are the imaginary part and real part of the dielectric permittivity (complex dielectric constant $\varepsilon=\varepsilon^{'}-i\varepsilon''$), respectively.

For finger motion sensing, the band-shaped gel was fixed to an index finger with adhesive tape, and the capacitance vs finger motion was recorded in air or water. For acoustic wave sensing, the music (Go time, Mark Petrie, USA) was switched on and off every 20 s near the pre-stretched gel while the capacitance was recorded with the LCR meter. The capacitance changes of the gels in response to various pre-strains and different volumes of music were both studied. The capacitances were normalized with the capacitances after prestrain as the 100% values (C_0_). The background noise was removed in the data analysis.

For water flow sensing in aqueous environments, the band-shaped SHARK gel was fixed at the two ends underwater. Then, flows from a syringe needle (diameter ~ 0.5 mm) were discontinuously applied to the middle of the gel band. The velocities were controlled with an injection pump (WZS-50F6, Smiths Medical, USA) connected to the syringe needle.

For the stress-relaxation experiments, the hydrogel samples were quickly preloaded to different strains in ~2-4 s, which were maintained for 120 s while the force and capacitance were synchronously recorded.

**Analogue simulation**

The analogue simulation was performed with ANSYS Workbench. The fluent and static structural modules from the workbench were accordingly used to perform the fluid-solid coupled simulation. The fluid simulation was first performed with the fluent module, and then, the resulting stress structures were applied to the static model. The fluid used in the fluent module was pure water, while the turbulence intensity and hydraulic diameter were set as 2% and 0.25 mm, respectively. A Young’s modulus of 28 kPa and a Poisson’s ratio of 0.48 were used in the static simulation. The two ends of the gel were fixed without considering the effects of gravity.

**Electromechanical self-healing tests**

For the test of the initial and self-healed SHARK band in a continuous test, the stretched gel was cut with a sharp plastic knife. Then, the freshly cut surfaces were brought back together such that they crossed over slightly and compressed for seconds before the stretching was continued (Figure 6b). For the time-dependent mechanical self-healing test, the freshly cut surfaces were brought back into contact and compressed for different times.

For the electrical self-healing test, the healing process was performed as described above under the monitoring of the LCR meter (HIOKI-IM3536, Japan). The freshly cut surfaces were brought back into contact and compressed for different times (2, 5, 10, 15 and 20 s) with various pressures (5, 12.5, 25 and 50 kPa) (Figure 6d and e). For the contact recovery of the capacitance (Figure S21), the freshly cut surfaces were continuously brought back into contact and disconnected using the tensile-compressive tester (Instron-5944, USA)

**Rheological measurements**

The hydrogels were carefully transferred to the rheometer plate of a Thermo Scientific Haake RheoStress 6000 (geometry: 1°/20 mm cone and plate; gap: 0.05 mm) with a spatula prior to the measurement. During the measurement, silicone oil was used to carefully cover the gap to avoid evaporation, and the temperature was controlled at 20 °C. For the strain thinning measurements, the viscosity was recorded in the strain range of 0.1 to 1000% at the oscillation frequency of 1 Hz. For the shear thinning measurements, the viscosity was recorded in the shear rate range of 0.1 to 100 s^-1^ at an oscillation strain of 1%. For the destruction-recovery measurements, G’ and G’’ were first monitored at an oscillation frequency of 1 Hz and an oscillation strain of 1%. Then, the strain and frequency were set to 500% and 100 Hz to destroy the crosslinking and switched back to a strain of 1% and a frequency of 1 Hz to monitor recovery of the mechanical properties.

**Remoulding and 3D printing of SHARK**

In the remoulding process of SHARK, the gel was pressed into different moulds directly, and gels with different shapes were obtained. For the measurements of the mechanical and electrical properties of remoulded SHARK, the gel was randomly compressed and remoulded into band-shaped shapes. 3D printing of SHARK was conducted based on a commercialized 3D printer (Bio-Architect@ WS, Regenovo, China) with a 210-μm nozzle. Typically, SHARK at a PCG concentration of 4.5 mg mL^-1^ was prepared as described above and directly printed into a square with an edge size of 15 mm and 1 layer, 2 layers, or 25 layers. To explore the printability of the hydrogel, several inner structures were employed to fulfill the square, including a grid path with θ=0° and 90°, a T-style path, a sine wave path, a radiation path, and a hexagon path. The pumping air stress, moving speed of the nozzle and layer thickness were set as 0.30 MPa, 4.0 mm s^-1^ and 200 μm, respectively. The distance between two filaments was set to 1200 μm.

**Figures**


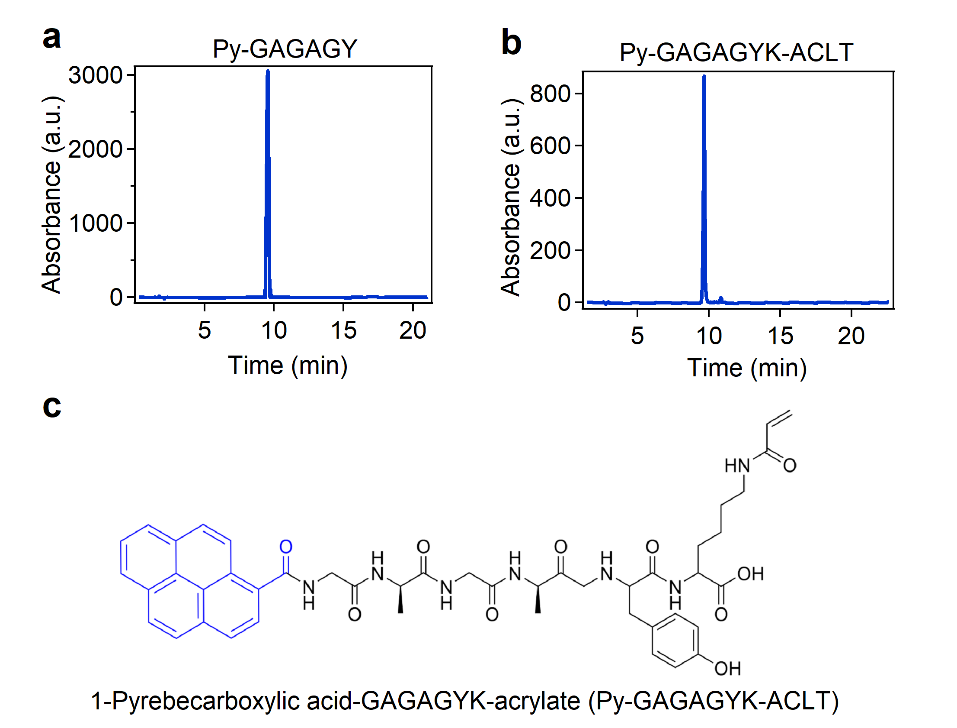


**Figure S1** **a-b**) HPLC traces for Py-GAGAGY (**a**) and Py-GAGAGYK-ACLT (**b**) peptides. **c**) Chemical structure of the Py-GAGAGYK-ACLT peptide.


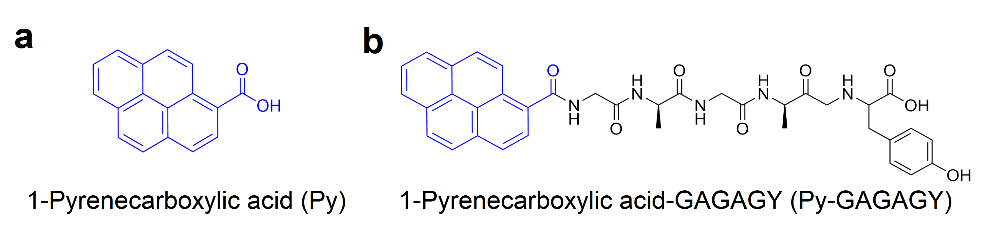


**Figure S2** Chemical structures of 1-pyrenecarboxylic acid (**a**) and 1-pyrenecarboxylic acid connected to the GAGAGY peptide (**b**).


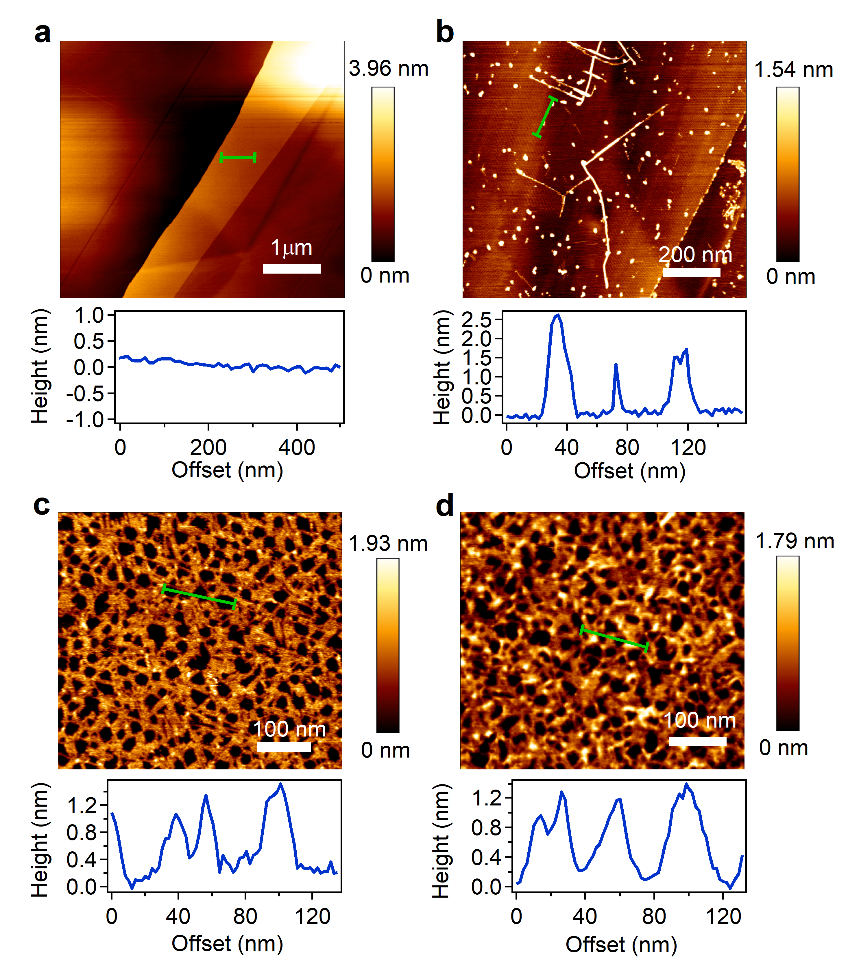


**Figure S3** AFM images of the self-assembled peptides on HOPG surfaces. **a**) AFM topography image (top) and height profile (bottom) of HOPG. **b)** AFM topography image (top) and height profile (bottom) of Py on HOPG. **c)** AFM topography image (top) and height profile (bottom) of Py-GAGAGY on HOPG. **d)** AFM topography image (top) and height profile (bottom) of Py-GAGAGY and Py-GAGAGY-mPEG on HOPG at a molar ratio of 10:1.


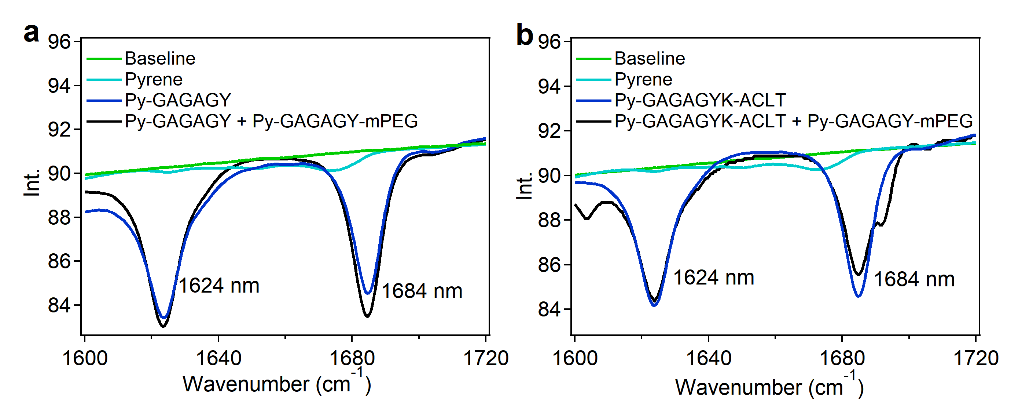


**Figure S4** FT-IR spectroscopy of the self-assembled peptides. **a)** FT-IR spectra of the self-assembled Py-GAGAGY peptide and co-assembly of Py-GAGAGY and Py-GAGAGY-mPEG. **b)** FT-IR spectra of the self-assembled Py-GAGAGYK-ACLT peptide and co-assembly of Py-GAGAGYK-ACLT and Py-GAGAGY-mPEG.


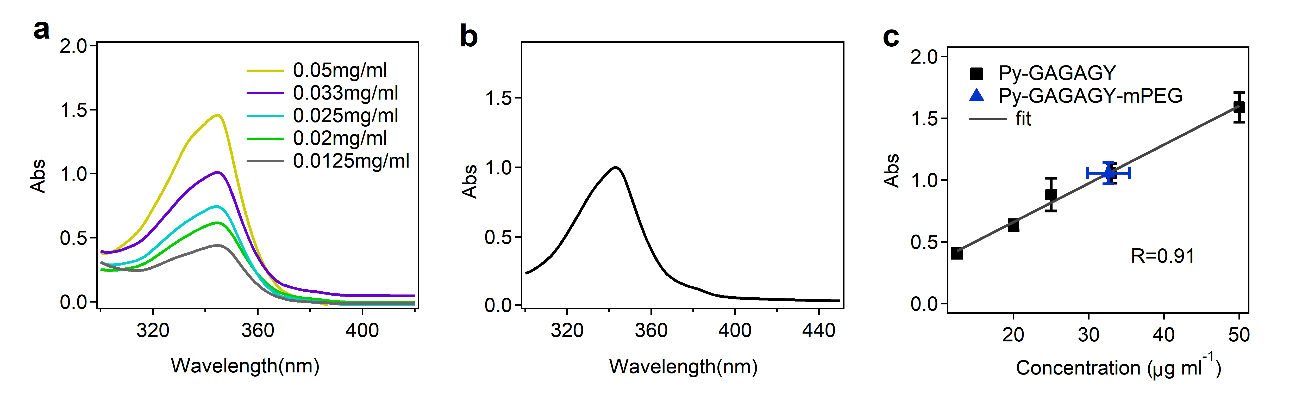


**Figure S5** Reaction efficiency of Py-GAGAGY and mPEG. **a)** UV-vis spectra of Py-GAGAGY peptides at various concentrations (0.0125-0.05 mg mL^-1^). **b)** UV-vis spectra of Py-GAGAGY-mPEG at a concentration of 0.5 mg mL^-1^. **c)** Calibration curve of Py-GAGAGY peptide concentrations based on UV absorbance at 344 nm. The blue point indicates the OD_344 nm_ of Py-GAGAGY-mPEG (0.5 mg mL^-1^) and the corresponding Py-GAGAGY concentration. The reaction efficiency of PEG and Py-GAGAGY reached 99.0%. Error bars indicate the mean ± S.D.


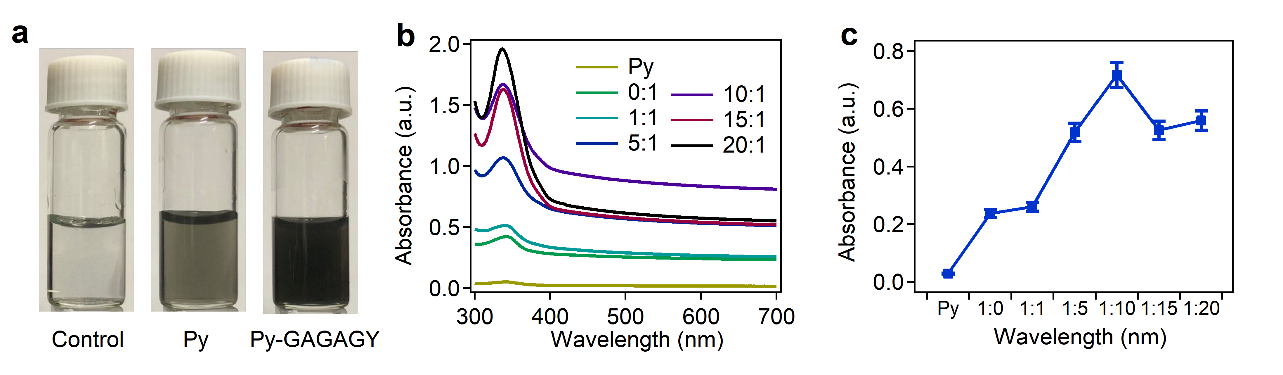


**Figure S6** Yields of PCG dependent on the molar ratio of Py-GAGAGY and Py-GAGAGY-mPEG. When optimizing the Py-GAGAGY:Py-GAGAGY-mPEG ratio, the concentration of Py-GAGAGY-mPEG remained fixed and the concentration of Py-GAGAGY was increased gradually. At low Py-GAGAGY:Py-GAGAGY-mPEG ratios, the peptide with bulky mPEG tails cannot assemble properly on graphene surfaces due to the steric hindrance. However, at high Py-GAGAGY:Py-GAGAGY-mPEG ratios, there were not sufficient PEG chains linked to the graphene surfaces. **a)** Optical images of obtained PCG dispersions in the presence of Py (middle) or Py-GAGAGY/Py-GAGAGY-mPEG (right, molar ratio of Py-GAGAGY and Py-GAGAGY-mPEG = 10:1). Dispersions obtained without any additives were set as the control group (left). **b)** UV spectra of PCG dispersions prepared with different molar ratios of Py-GAGAGY and Py-GAGAGY-mPEG (0:1, 1:1, 5:1, 10:1, 15:1, and 20:1). **c)** UV absorbance of PCG dispersions corresponding to Figure b at 660 nm. Error bars indicate the mean ± S.D.


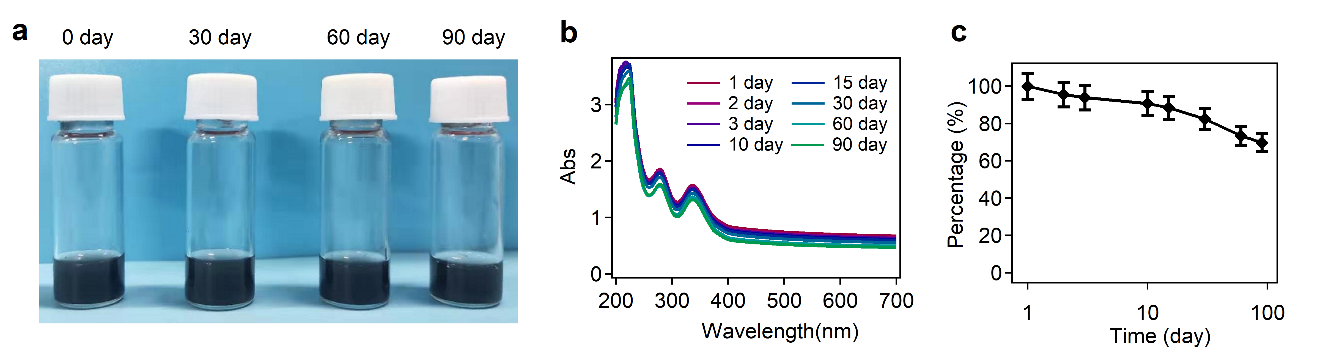


**Figure S7** Long-term stability of the PCG dispersion. **a)** Optical pictures of obtained PCG solutions stored at room temperature for different times. **b)** Long-term stability of the obtained PCG solutions, as estimated by UV/Vis spectroscopy. The barely changed absorbance at 214 nm indicated the ignorable degradation of peptide. **c)** Normalized PCG percentage remaining in the dispersions at 90 days. Error bars indicate the mean ± S.D.


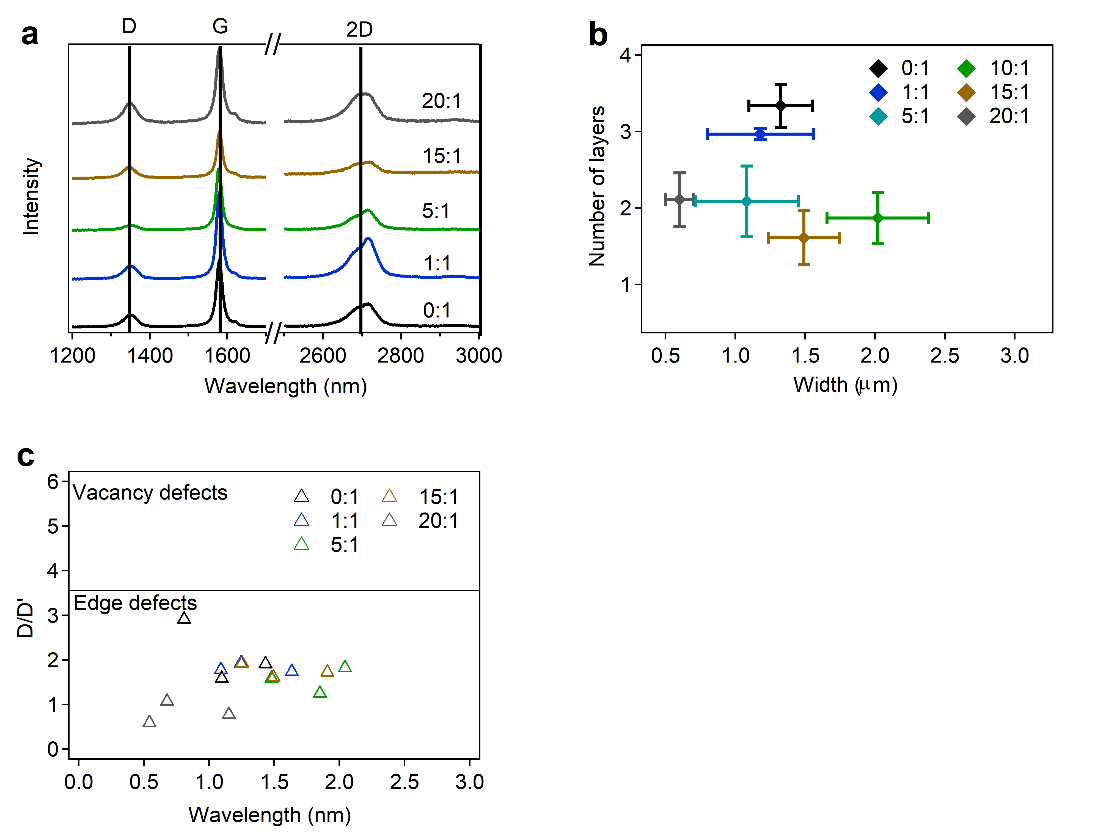


**Figure S8** Raman spectroscopy and defect analysis of PCG prepared with various ratios of Py-GAGAGY and Py-GAGAGY-mPEG (0:1, 1:1, 5:1, 10:1 and 20:1). **a)** Typical Raman spectra of graphene (excited at 532 nm). **b)** Statistical distributions of the layer number and graphene width for various Py-GAGAGY-mPEG and Py-GAGAGY ratios. **c)** Statistical distributions of D/D’ from the Raman spectra for PCG, which is a critical parameter of defects for graphene. Error bars indicate the mean ± S.D.


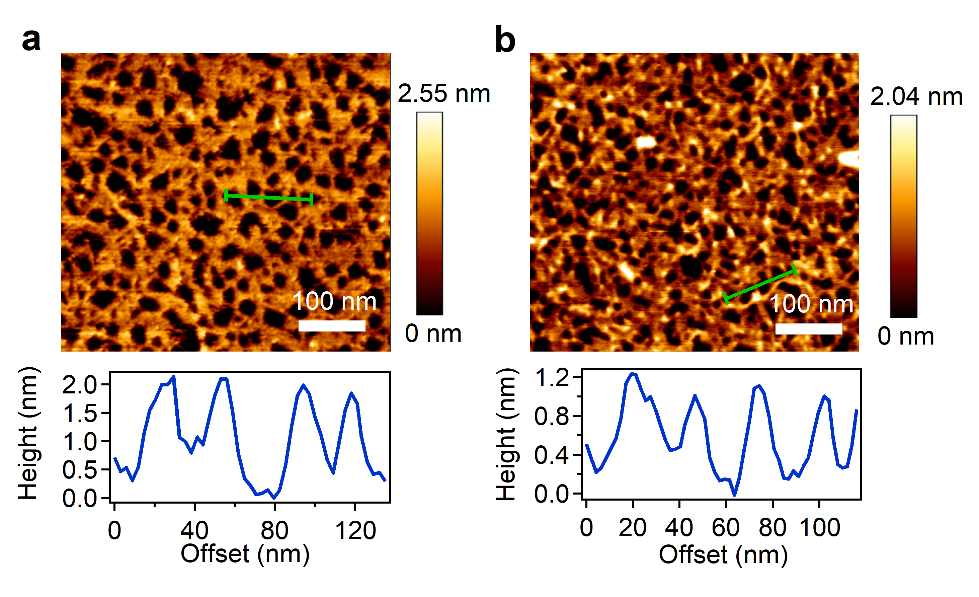


**Figure S9** AFM images of the self-assembly of acrylate-terminated peptides on HOPG surfaces. **a)** AFM topography image (top) and height profile (bottom) of Py-GAGAGYK-ACLT on HOPG. **b)** AFM topography image (top) and height profile (bottom) of Py-GAGAGYK-ACLT and Py-GAGAGY-mPEG (10:1) on HOPG. As acrylate moiety is also hydrophobic and can interaction with graphite surfaces, the self-assembled Py-GAGAGYK-ACLT on HOPG surfaces show a fibrous network structure instead of dispersed fibers as the Py-GAGAGYK on graphene surfaces. In this case both the N-terminus and the C-terminus of the peptides are layed on the graphite surface. Without the acrylate group, only the N-terminus of the peptides is on the graphene surface. Therefore the heights of the two fibers are different.


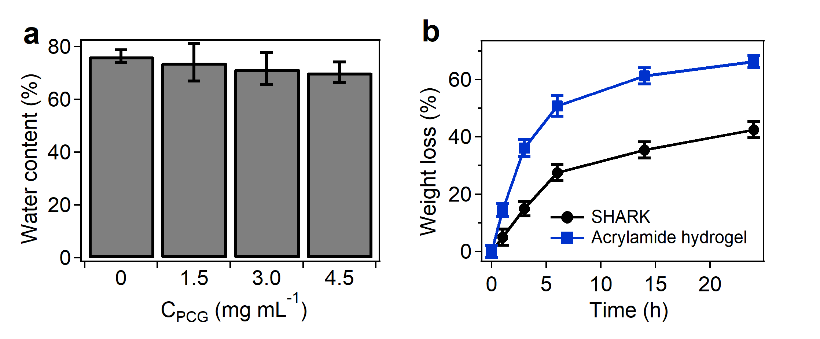


**Figure S10** Water contents and water evaporation of SHARK. (A) Water contents of SHARK at various concentrations of PCG (0, 1.5, 3.0 and 4.5 mg mL^-1^). (B) Weight loss vs time of SHARK and acrymalide hydrogel at the same mass concentrations at the humidity of ~40% and temperature of ~25 °C.


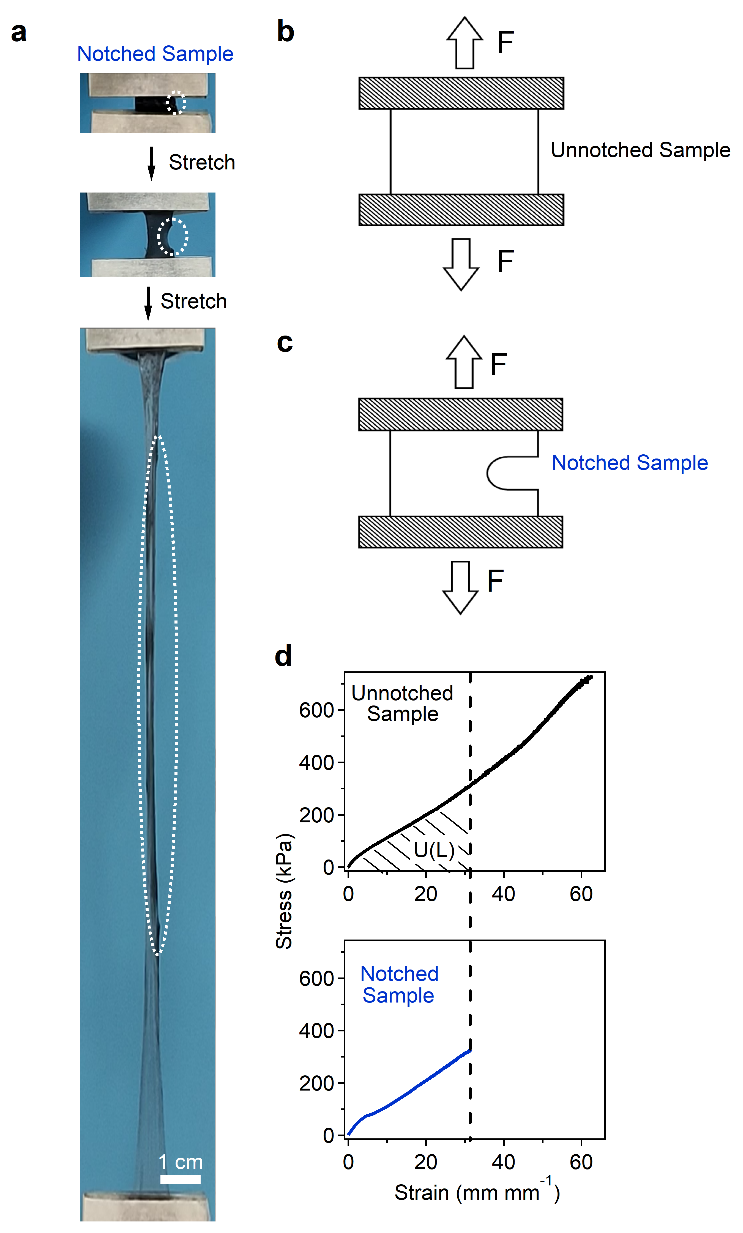


**Figure S11** Determination of the fracture energy. **a)** Experimental images of the fracture energy test. The upper clamp was pulled upward at a constant velocity of 10 mm min^-1^ away from the initial distance (L_0_ = 2.5 mm) between the two clamps, while the lower clamp was fixed. **b**, **c)** Schematic of the measurement of unnotched (**b**) and notched (**c**) samples. **d)** Stress-strain curves of unnotched (top) and notched (bottom) samples.


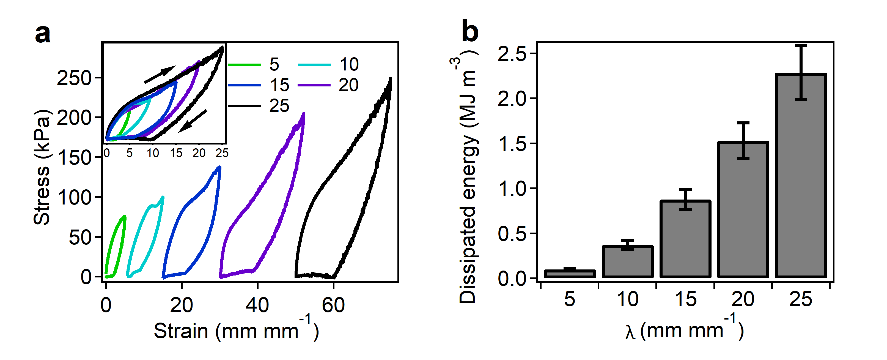


**Figure S12** Mechanical hysteresis of SHARK. **a)** Stretching-relaxation curves of SHARK (C_PCG_ = 4.5 mg mL^-1^) subjected to various maximum strains (λ = 5, 10, 15, 20 and 25 mm mm^-1^). Due to the difference of each hydrogel sample in preparation, the yield stress varied in a range of ~70 to 90 kPa. **b)** Summarized dissipated energy of SHARK at different strains corresponding to the cycles in **a**.


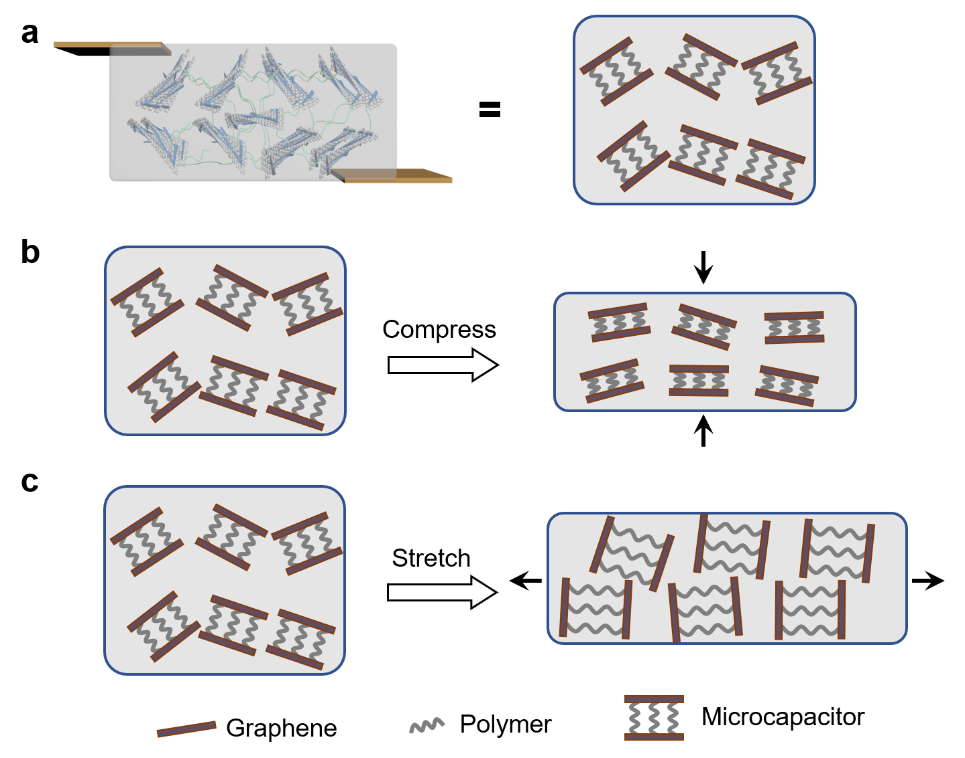


**Figure S13** Structure and the sensing mechanism of SHARK. **a)** Simplified schematic of the structure for SHARK. **b)** Schematic for compression sensing mechanism of SHARK. **c)** Schematic for stretch sensing mechanism of SHARK.


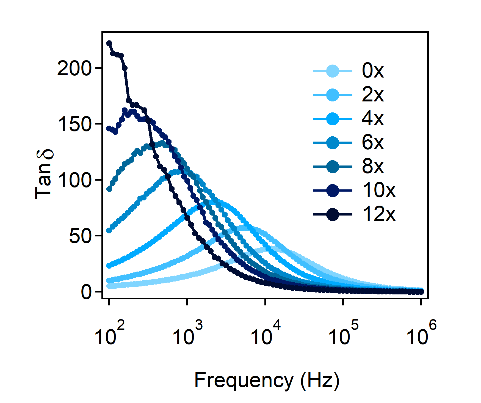


**Figure S14** Frequency response of the dielectric loss (tan δ) of SHARK at various strains (0, 2, 4, 6, 8, 10 and 12 mm mm^-1^). The frequency response peak shifted to the left with the increasing strain.


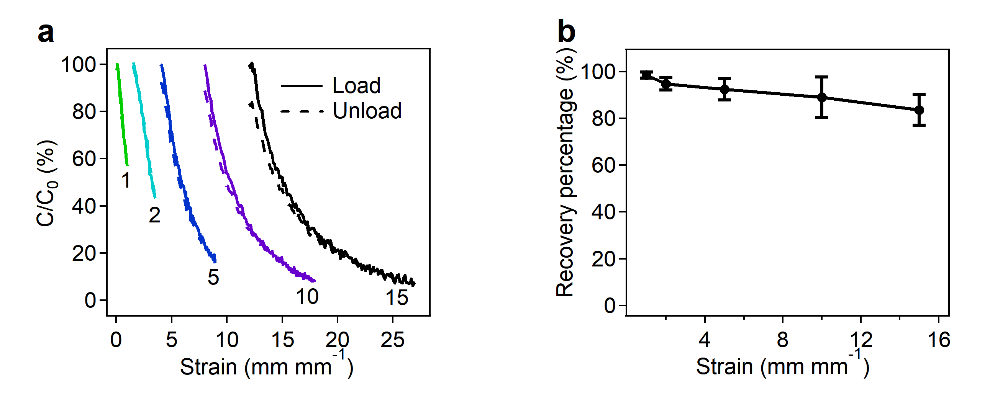


**Figure S15** Hysteresis and normalized recovery of the capacitance at various strains. **a)** Hysteresis of the capacitance when subjected to different strains. **b)** Capacitance recovery when subjected to different strains.


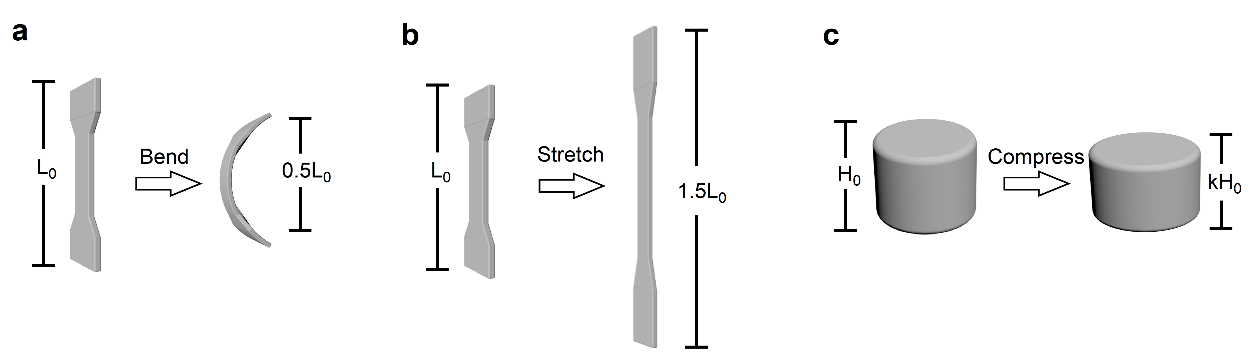


**Figure S16** Schematic of the strain and pressure sensing using SHARK. **a**, **b)** Bending (**a**) and stretching (**b**) sensing schematics of band-shaped SHARK. **c)** Pressure sensing schematic of disc-shaped SHARK.


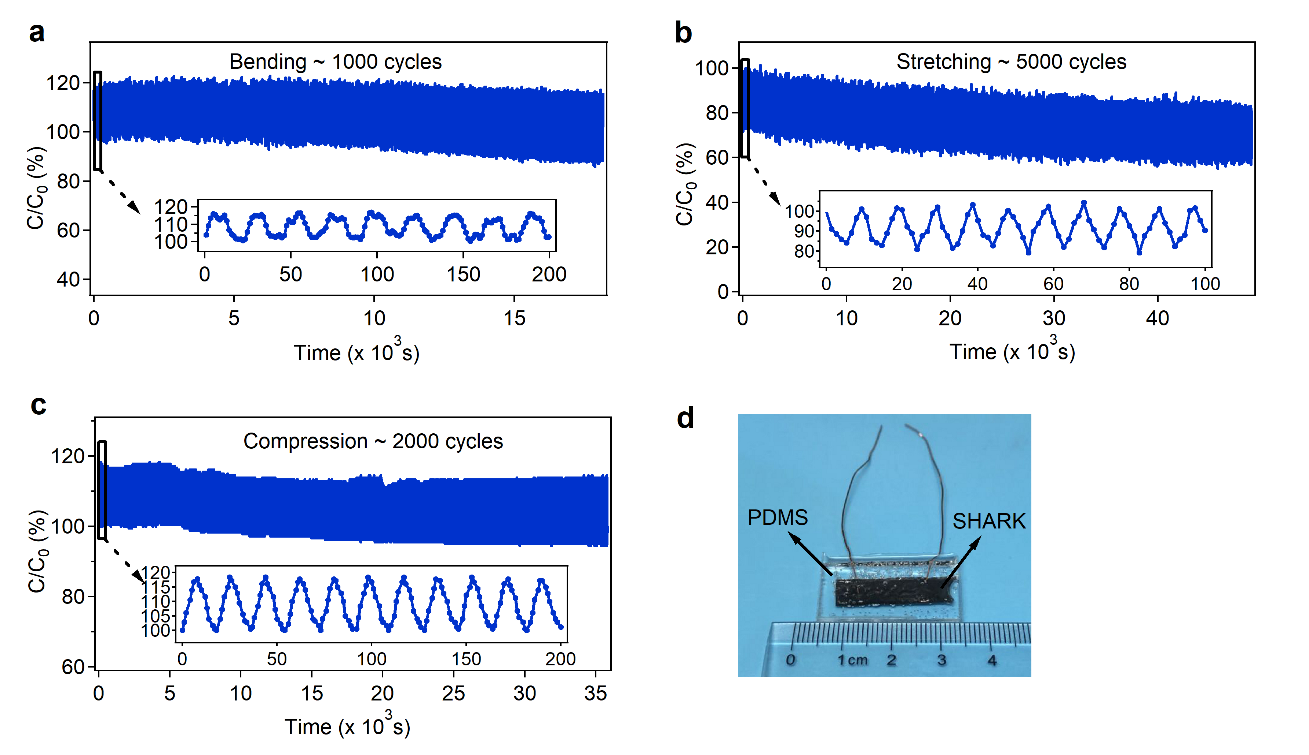


**Figure S17** Change in the capacitance of the PDMS-coated SHARK in 1000 bending cycles (**a**), 5000 stretching cycles (**b**) and 2000 compression cycles (**c**). There is no holding time in the stretching and compression cycles to achieve more motion cycles in a shorter time. **d**) Optical image of the typical PDMS-coated SHARK.


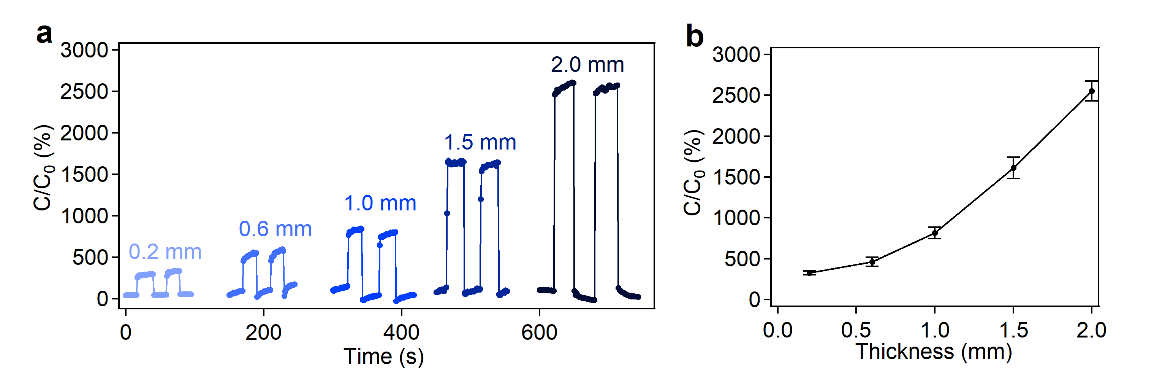


**Figure S18** Effects of the thickness of SHARK on the finger bending sensing in air. **a)** Relative capacitance changes of band-shaped SHARK at different thickness for finger bending in two states (0.2-2.0 mm). **b)** Capacitance change amplitudes correspond to band-shaped SHARK at different thickness (0.2-2.0 mm).


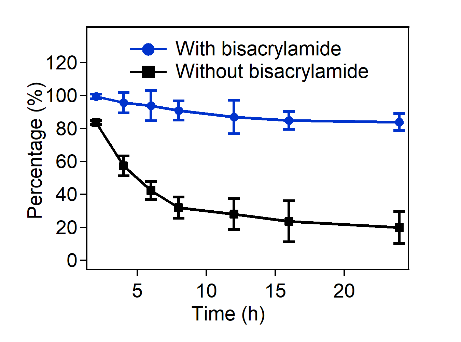


**Figure S19** Evaluation of erosion for SHARK with and without bis-acrylamide (0.5% w/v) in water by monitoring the dry weight of the hydrogels in 24 hours.


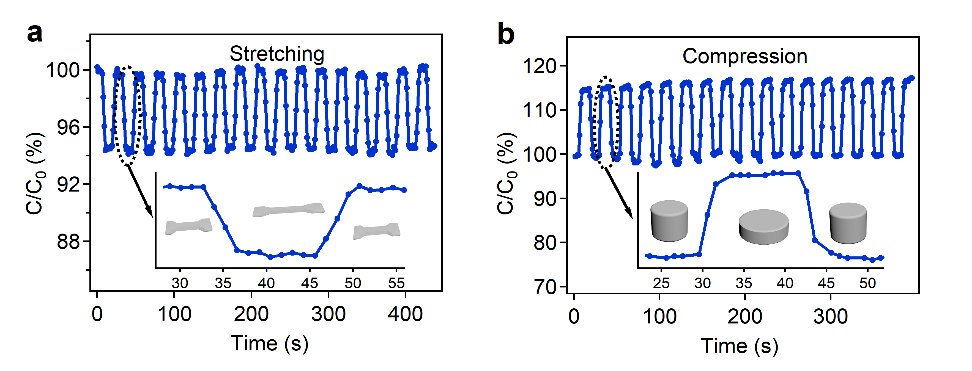


**Figure S20** Simple motion sensing of SHARK in water. **a)** Change in the capacitance of the band-shaped SHARK sensor in multiple stretching cycles at a strain of 0.5 mm mm^-1^ in water. **b)** Change in the capacitance of the column-shaped SHARK sensor in multiple compression cycles at a strain of 0.15 mm mm^-1^ in water.


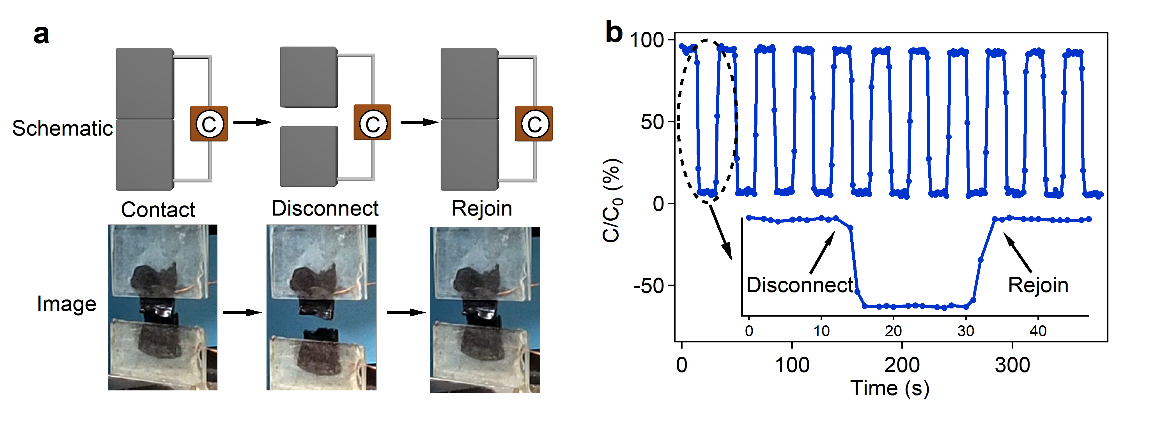


**Figure S21** Demonstration of the healing process of the capacitance for SHARK upon rejoining. **a)** Schematic and images of the capacitance healing process upon rejoining. **b)** Normalized capacitance healing in continuous disconnection and rejoining cycles


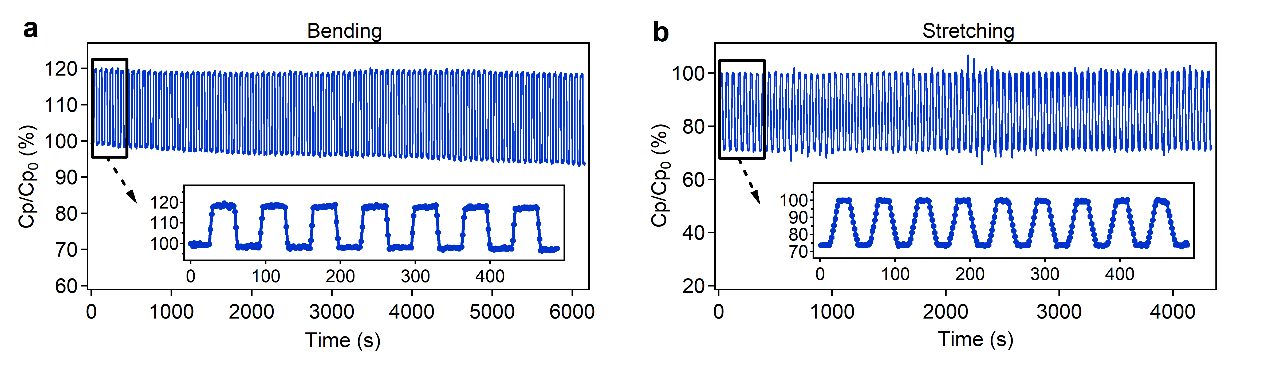


**Figure S22** Demonstration of simple motion sensing of SHARK after 10 cycles of healing/cutting. **a)** Change in the capacitance of the band-shaped SHARK sensor in multiple bending cycles at a strain of 0.5 mm mm^-1^. **b)** Change in the capacitance of the column-shaped SHARK sensor in multiple stretching cycles at a strain of 0.5 mm mm^-1^.


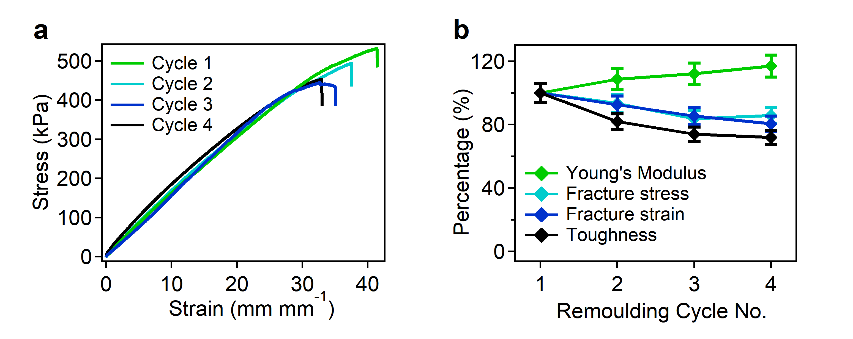


**Figure S23** Mechanical properties of SHARK after remoulding. **a)** Uniaxial stress-strain curves of the same hydrogel after being remoulded 1, 2, 3 and 4 times. **b)** Normalized Young’s modulus, fracture strain, fracture stress and toughness of hydrogels with different remoulding times. The hydrogel remoulded 1 time was set as the 100% group.


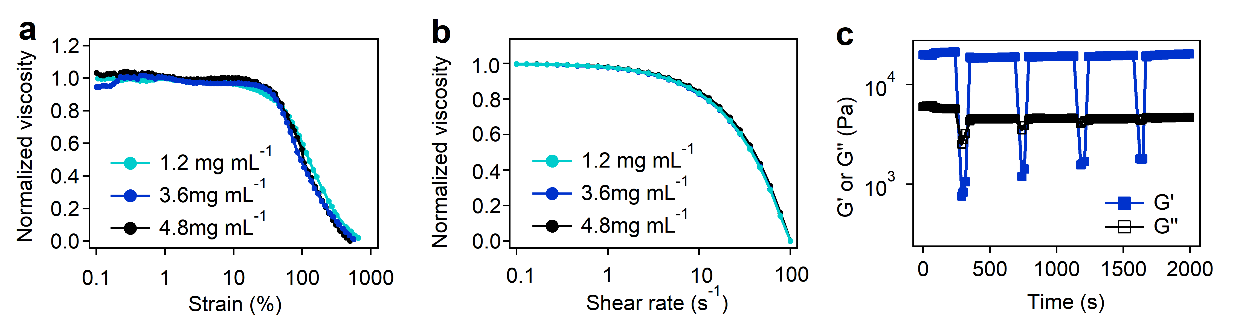


**Figure S24** Strain thinning, shear thinning and fast recovery properties of SHARK measured with a rheometer. **a**, **b)** Strain thinning (**a**) and shear thinning (**b**) of the SHARK hydrogel. The viscosity decreased with increasing strain or shear rate. **c)** Fast microscopic recovery of SHARK (C_PCG_ = 4.5 mg mL^-1^) observed in the consecutive destruction-recovery measurements with a rheometer. The strain and frequency were set to 500% and 100 Hz to destroy the weak interactions and switched back to an amplitude of 1% and a frequency of 1 Hz to monitor recovery of the mechanical properties.

**Table S1**. Summary of the yields from graphene production by ultrasonication in aqueous conditions with different biomacromolecules as the stabilizer.

| Stabilizer | Exfoliation method | | Yield (mg mL^-1^) and productivity | Lateral size (μm) | Thickness (nm) | Exfoliation condition |
| --- | --- | --- | --- | --- | --- | --- |
| **Py-GAGAGY peptide (this work)** | **Tip ultrasonication** | | **0.64 (1.87)** | **1.5-2.5** | **1-3** | **C_1_: 1.0 (2.5) mg mL^-1^; 3 h** |
| Gelatine (11) | Bath ultrasonication | | 0.6 | 0.57 | 2.19 | C_1_ : 100 mg mL^-1^; 80 h |
| Pyrene derivatives (12) | | Tip ultrasonication | 0.8-1 | N/A | 2-4 layers | C_1_: 6 mg mL^-1^;7 W, 1 h |
| Pullulan (13) | | Tip ultrasonication | 2.3 | N/A | < 5 layers | C_1_: 50 mg mL^-1^; 200 W, 10-60 min |
| Chitosan (14) | | Tip ultrasonication | 5.5 | 10-15 nm | N/A | C_1_: 20 mg mL^-1^; 200 W, 10-60 min |
| DNA (14) | | Tip ultrasonication | 2.29 | 235 nm | 1.3 nm | C_1_: 10-150 mg mL;750 W, 6 h |
| Gum arabic (15) | | Bath ultrasonication | 0.5-0.6 | N/A | N/A | C_1_: 10 mg mL^-1^ ;100 h |
| Sodium cholate (NaC) (16) | | Bath ultrasonication | 0.05-0.3 | 0.3 - 1.2 | mono-layer | C_1_: 5 mg mL^-1^ ; 400 h |
| Graphene oxide (17) | | Ultrasonication | N/A | < 1 | 1-5 layers | C_1_: 1.5 mg mL^-1^; 150 W, 10 h |
| Sodium citrate (18) | | Ultrasonication | 0.71 | < 1 | 1.5 nm, few-layer | C_1_: 10 mg mL^-1^; 1000 W, 4 h |

**Table S2.** Mechanical properties of SHARK containing different concentrations of PCG. Data are presented as the average ± S.D. *

| C_acrylamide_  (mg mL^-1^) | C_PCG_ (mg mL^-1^) | Tensile strain limit  (mm mm^-1^) | Fracture stress  (MPa) | Young’s modulus (kPa) | Toughness  (MJ m^-3^) |
| --- | --- | --- | --- | --- | --- |
| 225 | 0 | 6.01 ± 0.81 | 0.015 ± 0.002 | 9.11 ± 2.42 | 0.13 ± 0.02 |
|  | 1.5 | 19.25 ± 2.25 | 0.142 ± 0.018 | 18.72 ± 3.32 | 1.33 ± 0.15 |
|  | 3.0 | 42.2 ± 6.20 | 0.382 ± 0.043 | 20.49 ± 5.64 | 7.46 ± 1.06 |
|  | 4.5 | 77.36 ± 8.18 | 1.02 ± 0.11 | 28.82 ± 5.12 | 32.64 ± 7.81 |

**Table S3**. Mechanical properties of SHARK and other kinds of stretchable and tough hydrogels.

| **Classification** | **Sample code** | **Young’s modulus (MPa)** | **Fracture strain (mm mm^-1^)** | **Fracture strength (MPa)** | **Toughness** **(MJ m^-3^)** | **Fracture energy** **(kJ m^-2^)** |
| --- | --- | --- | --- | --- | --- | --- |
| **Single network hydrogel** | CB[8]-PAM (19) | 0.02-0.42 | 107 | 1.8 | -- | -- |
|  | PAM-peptide-Zn^2+^ (20) | 0.01-0.12 | 4.3-7.8 | 0.2-0.56 | -- | 0.63-1.35 |
|  | P(NaSS-co-MPTC) (7) | 1.53 | 9.4 | 2.6 | -- | 4 |
|  | DMAA-co-MAAc (21) | 28 | ~8 | 2 | -- | 9.3 |
| **Double network hydrogel** | Alginate/PAAm (8) | 0.029 | 23 | 0.156 | -- | 8.7 |
|  | PS-DN (22) (compression) | 0.32-0.57 | -- | 0.21-1.6 | -- | 0.3-2.67 |
|  | Crystallized PVA/PAAm (23) | 5 | ~3.8 | 2.5 | -- | 14 |
|  | Agar/PAAm (24) | 0.082 | 20 | 1 | 9 | -- |
|  | Agar/HPAAm (25) | 0.106 | 52.6 | 0.267 | 9.35 | 1 |
| **Nanocomposite hydrogel** | **SHARK (this work)** | **0.028** | **77** | **1.02** | **32.6** | **19.75** |
|  | NPs-P-PAA (26) | ~0.02 | 26 | 0.11 | -- | 5.5 |
|  | Oxidized CNT/PAACA (27) | 0.028 | 14.1 | 0.364 | -- | -- |
|  | PDA-pGO-PAM (28) | ~0.01 | >35 | ~0.17 | -- | 6.78 |
|  | P(BMA-co-AA)/PAM (29) | 0.028 | 17.4 | 0.74 | ~6 | -- |
|  | (PDDA/PEI)-(PSS/PAA) (30) | 0.36 ± 0.03 | 24.3 | 1.26 | 19.53 | -- |
| **Other hydrogel** | NIPA-AAcNa-HPR-C (31) | ~0.01 | ~15 | ~0.033 | -- | -- |
|  | Carboxyl-Fe^3+^/PR (32) | 8.3 | 5 | 4 | -- | 10 |
|  | Tetra-PEG (33) | 0.054 | 7.4 | 0.136 | 0.59 | -- |
|  | Tetra-PEG protein (34) | 0.027 | 2.5 | 0.035 | -- | -- |

**Table S4**. Comparison of SHARK and recently reported capacitive strain sensors.

| **Materials** | **Self-healability** | **Sensing limit** | **Sensitivity** |
| --- | --- | --- | --- |
| **SAHRK (this work)** | **Yes (in seconds)** | **2600%** | **1.39 (1.49)** |
| P(AAc-co-SBMA) (35) | No | 100% | 1 |
| ACC/PAA/alginate (36) | No |  | 1 |
| PAAm/NaCl (37) | No |  | 0.65 |
| MXene (Ti_3_C_2_)/ Polyvinyl  alcohol (PVA) (38) | Yes (0.15 s) | 200% | 0.4 |
| Au- Polyethylene terephthalate (PET) (39) | No | 140 % | 3.05 |
| Carbon Nanotubes (CNTs)-Silicone elastomer (40) | No | 100 % | 0.99 |
| Block copolymer (BCP) structural color (SC): Ionic gel (IG) film-PDMS (41) | No | 100 % | 0.6 |
| Ionically conductive fluid (sodium chloride glycerol- polyethylene glycol)-Silicone elastomer (42) | No | 250 % | 0.348 ± 11 |
| Fabric-Silicone (43) | No | 100 % | 1.23 |
| AgNWs-Ecoflex (44) | No | 50 % | 0.7 |
| PDMS-MDU0.4-IU0.6 film (45) | Yes(12 hr) | 100 % | 1 |
| CNTs-Ecoflex (46) | No | 150 % | 1 |
| AgNW/PDMS-MPU0.4-IU0.6 (47) | Yes (12 hr) | 50 % | - |
| LM (EGaIn)-hollowelastomeric fibers (48) | No | 100 % | 0.66-0.82 |

**Movie S1.**

Blowing the SHARK balloon with argon gas.

**Movie S2.**

Stretching a band-shaped SHARK to 77 times its original length.

**Movie S3.**

Stretching a notched band-shaped SHARK.

**Movie S4.**

Capacitive response of SHARK fixed on a human finger upon bending in three states.

**Movie S5.**

Capacitive response of SHARK upon reversibly turning the music on and off at a pre-strain of 40 mm mm^-1^.

**Movie S6.**

Capacitive response of SHARK to different sound intensities at a pre-strain of 40 mm mm^-1^.

**Movie S7.**

Cutting, rejoining and stretching of SHARK.

**Movie S8.**

Real-time healing of SHARK at a pre-strain of 73 mm mm^-1^.

**Movie S9.**

Real-time restoring electrical properties of SHARK upon touching.

**Movie S10.**

3D extrusion printing of SHARK.

**References:**

1. Evans, E, Ritchie, K. Strength of a weak bond connecting flexible polymer chains. *Biophys J* 1999; **76**(5): 2439-47.

2. Bell, GI. Models for the specific adhesion of cells to cells. *Science* 1978; **200**(4342): 618.

3. Lotya, M, Hernandez, Y, King, PJ*, et al.* Liquid phase production of graphene by exfoliation of graphite in surfactant/water solutions. *J Am Chem Soc* 2009; **131**(10): 3611-20.

4. Paton, KR, Varrla, E, Backes, C*, et al.* Scalable production of large quantities of defect-free few-layer graphene by shear exfoliation in liquids. *Nat Mater* 2014; **13**(6): 624-30.

5. Berkdemir, A, Gutierrez, HR, Botello-Mendez, AR*, et al.* Identification of individual and few layers of WS2 using Raman Spectroscopy. *Sci Rep* 2013; **3**(1): 1755.

6. Zeng, H, Liu, GB, Dai, J*, et al.* Optical signature of symmetry variations and spin-valley coupling in atomically thin tungsten dichalcogenides. *Sci Rep* 2013; **3**(1): 1608.

7. Sun, TL, Kurokawa, T, Kuroda, S*, et al.* Physical hydrogels composed of polyampholytes demonstrate high toughness and viscoelasticity. *Nat Mater* 2013; **12**(10): 932-7.

8. Sun, JY, Zhao, X, Illeperuma, WR*, et al.* Highly stretchable and tough hydrogels. *Nature* 2012; **489**(7414): 133-6.

9. Tanaka, Y, Kuwabara, R, Na, YH*, et al.* Determination of fracture energy of high strength double network hydrogels. *J Phys Chem B* 2005; **109**(23): 11559-62.

10. Jackson, AP. Measurement of the fracture toughness of some contact lens hydrogels. *Biomaterials* 1990; **11**(6): 403-7.

11. Ge, Y, Wang, JL, Shi, ZX*, et al.* Gelatin-assisted fabrication of water-dispersible graphene and its inorganic analogues. *J Mater Chem* 2012; **22**(34): 17619-24.

12. Parviz, D, Das, S, Ahmed, HS*, et al.* Dispersions of non-covalently functionalized graphene with minimal stabilizer. *ACS Nano* 2012; **6**(10): 8857-67.

13. Uysal Unalan, I, Wan, C, Trabattoni, S*, et al.* Polysaccharide-assisted rapid exfoliation of graphite platelets into high quality water-dispersible graphene sheets. *RSC Adv* 2015; **5**(34): 26482-90.

14. Joseph, D, Seo, S, Williams, DR*, et al.* Double-stranded DNA-graphene hybrid: preparation and anti-proliferative activity. *ACS Appl Mater Inter* 2014; **6**(5): 3347-56.

15. Chabot, V, Kim, B, Sloper, B*, et al.* High yield production and purification of few layer graphene by gum arabic assisted physical sonication. *Sci Rep* 2013; **3**(1): 1378.

16. Lotya, M, King, PJ, Khan, U*, et al.* High-concentration, surfactant-stabilized graphene dispersions. *ACS Nano* 2010; **4**(6): 3155-62.

17. Tung, TT, Yoo, J, Alotaibi, FK*, et al.* Graphene Oxide-Assisted Liquid Phase Exfoliation of Graphite into Graphene for Highly Conductive Film and Electromechanical Sensors. *ACS Appl Mater Inter* 2016; **8**(25): 16521-32.

18. Li, J, Yan, H, Dang, D*, et al.* Salt and water co-assisted exfoliation of graphite in organic solvent for efficient and large scale production of high-quality graphene. *J Colloid Inter Sci* 2019; **535**: 92-9.

19. Liu, J, Tan, CSY, Yu, Z*, et al.* Tough Supramolecular Polymer Networks with Extreme Stretchability and Fast Room-Temperature Self-Healing. *Adv Mater* 2017; **29**(22): 1605325.

20. Zeng, L, Song, M, Gu, J*, et al.* A Highly Stretchable, Tough, Fast Self-Healing Hydrogel Based on Peptide–Metal Ion Coordination. *Biomimetics* 2019; **4**(2).

21. Hu, X, Vatankhah-Varnoosfaderani, M, Zhou, J*, et al.* Weak Hydrogen Bonding Enables Hard, Strong, Tough, and Elastic Hydrogels. *Adv Mater* 2015; **27**(43): 6899-905.

22. Sun, W, Xue, B, Li, Y*, et al.* Polymer-Supramolecular Polymer Double-Network Hydrogel. *Adv Funct Mater* 2016; **26:** 9044-9052.

23. Li, J, Suo, Z, Vlassak, JJ. Stiff, strong, and tough hydrogels with good chemical stability. *Journal of Materials Chemistry B* 2014; **2**(39): 6708-13.

24. Chen, Q, Zhu, L, Zhao, C*, et al.* A Robust, One-Pot Synthesis of Highly Mechanical and Recoverable Double Network Hydrogels Using Thermoreversible Sol-Gel Polysaccharide. *Adv Mater* 2013; **25**(30): 4171-6.

25. Chen, Q, Zhu, L, Chen, H*, et al.* A Novel Design Strategy for Fully Physically Linked Double Network Hydrogels with Tough, Fatigue Resistant, and Self-Healing Properties. *Adv Funct Mater* 2015; **25**(10): 1598-607.

26. Gan, D, Xing, W, Jiang, L*, et al.* Plant-inspired adhesive and tough hydrogel based on Ag-Lignin nanoparticles-triggered dynamic redox catechol chemistry. *Nat Commun* 2019; **10**(1): 1487.

27. Rehman, HU, Chen, Y, Guo, Y*, et al.* Stretchable, strong and self-healing hydrogel by oxidized CNT-polymer composite. *Compos Part A-Appl S* 2016; **90**: 250-60.

28. Zhang, Y, Hu, H, Pei, X*, et al.* Polymer brushes on structural surfaces: a novel synergistic strategy for perfectly resisting algae settlement. *Biomater Sci* 2017; **5**(12): 2493-500.

29. Xu, K, Liang, X, Li, P*, et al.* Tough, stretchable chemically cross-linked hydrogel using core–shell polymer microspheres as cross-linking junctions. *Polymer* 2017; **118**: 58-67.

30. Yuan, T, Cui, X, Liu, X*, et al.* Highly Tough, Stretchable, Self-Healing, and Recyclable Hydrogels Reinforced by in Situ-Formed Polyelectrolyte Complex Nanoparticles. *Macromolecules* 2019; **52**(8): 3141-9.

31. Bin Imran, A, Esaki, K, Gotoh, H*, et al.* Extremely stretchable thermosensitive hydrogels by introducing slide-ring polyrotaxane cross-linkers and ionic groups into the polymer network. *Nat Commun* 2014; **5**(1): 5124.

32. Zheng, SY, Liu, C, Jiang, L*, et al.* Slide-Ring Cross-Links Mediated Tough Metallosupramolecular Hydrogels with Superior Self-Recoverability. *Macromolecules* 2019; **52**(17): 6748-55.

33. Ishii, S, Kokubo, H, Hashimoto, K*, et al.* Tetra-PEG Network Containing Ionic Liquid Synthesized via Michael Addition Reaction and Its Application to Polymer Actuator. *Macromolecules* 2017; **50**(7): 2906-15.

34. Wu, J, Li, P, Dong, C*, et al.* Rationally designed synthetic protein hydrogels with predictable mechanical properties. *Nat Commun* 2018; **9**(1): 620.

35. Lei, Z, Wu, P. Zwitterionic Skins with a Wide Scope of Customizable Functionalities. *ACS Nano* 2018; **12**(12): 12860-8.

36. Lei, Z, Wang, Q, Sun, S*, et al.* A Bioinspired Mineral Hydrogel as a Self-Healable, Mechanically Adaptable Ionic Skin for Highly Sensitive Pressure Sensing. *Adv Mater* 2017; **29**(22): 1700321.

37. Sun, J-Y, Keplinger, C, Whitesides, GM*, et al.* Ionic skin. *Adv Mater* 2014; **26**(45): 7608-14.

38. Zhang, J, Wan, L, Gao, Y*, et al.* Highly Stretchable and Self-Healable MXene/Polyvinyl Alcohol Hydrogel Electrode for Wearable Capacitive Electronic Skin. *Adv Electron Mater* 2019; **5**(7): 1900285.

39. Nur, R, Matsuhisa, N, Jiang, Z*, et al.* A Highly Sensitive Capacitive-type Strain Sensor Using Wrinkled Ultrathin Gold Films. *Nano Lett* 2018; **18**(9): 5610-7.

40. Cohen, DJ, Mitra, D, Peterson, K*, et al.* A Highly Elastic, Capacitive Strain Gauge Based on Percolating Nanotube Networks. *Nano Lett* 2012; **12**(4): 1821-5.

41. Park, TH, Yu, S, Cho, SH*, et al.* Block copolymer structural color strain sensor. *NPG Asia Mater* 2018; **10**(4): 328-39.

42. Frutiger, A, Muth, JT, Vogt, DM*, et al.* Capacitive Soft Strain Sensors via Multicore–Shell Fiber Printing. *Adv Mater* 2015; **27**(15): 2440-6.

43. Atalay, A, Sanchez, V, Atalay, O*, et al.* Batch Fabrication of Customizable Silicone-Textile Composite Capacitive Strain Sensors for Human Motion Tracking. *Adv Mater Technol* 2017; **2**(9): 1700136.

44. Yao, S, Zhu, Y. Wearable multifunctional sensors using printed stretchable conductors made of silver nanowires. *Nanoscale* 2014; **6**(4): 2345-52.

45. Kang, J, Son, D, Wang, G-JN*, et al.* Tough and Water-Insensitive Self-Healing Elastomer for Robust Electronic Skin. *Adv Mater* 2018; **30**(13): 1706846.

46. Shin, U-H, Jeong, D-W, Park, S-M*, et al.* Highly stretchable conductors and piezocapacitive strain gauges based on simple contact-transfer patterning of carbon nanotube forests. *Carbon* 2014; **80**: 396-404.

47. Son, D, Kang, J, Vardoulis, O*, et al.* An integrated self-healable electronic skin system fabricated via dynamic reconstruction of a nanostructured conducting network. *Nat Nanotechnol* 2018; **13**(11): 1057-65.

48. Cooper, CB, Arutselvan, K, Liu, Y*, et al.* Stretchable Capacitive Sensors of Torsion, Strain, and Touch Using Double Helix Liquid Metal Fibers. *Adv Funct Mater* 2017; **27**(20): 1605630.
